# Transcriptomics and functional genomics implicate WNT3 in hemispheric lateralization of speech production

**DOI:** 10.1016/j.isci.2026.114692

**Published:** 2026-01-14

**Authors:** Zixian Wang, Yanxi Chen, Yongqi Feng, Gaoyu Zu, Wenxu Wang, Limiao Liang, Xinyu Liu, Yuhang Mei, Jiaxuan Yang, Tian Wang, Mengqi Liu, Linxi Jiang, Russell G. Snell, Blake Highet, Maurice Curtis, Liang Chen, Shi-Bin Li, Jinsong Wu, Menghan Zhang, Wensheng Li, Gong-Hong Wei, Linya You

**Affiliations:** 1 State Key Laboratory of Common Mechanism Research for Major Diseases, Suzhou Institute of Systems Medicine, Chinese Academy of Medical Sciences&Peking Union Medical College, Suzhou 215123, China; 2 MOE Key Laboratory of Metabolism and Molecular Medicine and Department of Biochemistry and Molecular Biology of School of Basic Medical Sciences&Fudan University Shanghai Cancer Center, Shanghai Medical College of Fudan University, Shanghai 200032, China; 3 Department of Human Anatomy&Histoembryology, School of Basic Medical Sciences, Fudan University, Shanghai 200032, China; 4 State Key Laboratory of Genetics and Development of Complex Phenotypes, Center for Evolutionary Biology, Human Phenome Institute, Zhangjiang Fudan International Innovation Center, School of Life Sciences, Fudan University, Shanghai, China; 5 Institute for Translational Brain Research, State Key Laboratory of Medical Neurobiology, MOE Frontiers Center for Brain Science, Fudan University, Shanghai, China; 6 HanGene Biotech, Xiaoshan Innovation Polis, Hangzhou, Zhejiang 31200, China; 7 Applied Translational Genetics Group, School of Biological Sciences, the University of Auckland, Auckland, New Zealand; 8 Department of Anatomy and Medical Imaging, The University of Auckland, Auckland, New Zealand; 9 Glioma Surgery Division, Neurological Surgery Department of Huashan Hospital, Fudan University, Shanghai 200040, China; 10 Research Institute of Intelligent Complex Systems and Institute of Modern Languages and Linguistics, Fudan University, Shanghai 200437, China; 11 Key Laboratory of Medical Imaging Computing and Computer Assisted Intervention of Shanghai, Shanghai 200032, China

**Keywords:** Audiology, Neuroscience, Sensory neuroscience, Genomics

## Abstract

Human speech and language depend on hemispheric specialization across cortical regions and cortico-striatal circuits. We profiled 125 human cortical samples from 13 Brodmann areas (BAs), bilaterally, across five donors to generate a hemisphere-resolved transcriptomic atlas and quantify region-specific lateralization. Integrating genome-wide association signals for speech-, language-, and reading-related traits with brain *cis*-expression quantitative trait loci (eQTL) and enhancer maps prioritized a regulatory axis linking rs62060948 to MYC binding and WNT3 expression. WNT3 was higher in right BA44 than left, and cellular assays supported MYC occupancy and showed reduced WNT3 after MYC knockdown. In mice, unilateral Wnt3 overexpression (*Wnt3* OE) in the dorsal striatum selectively altered ultrasonic vocalizations (USVs), locomotor activity, and myelin basic protein expression. These results connect regulatory variation to lateralized gene control and circuit function relevant to vocal communication, and provide a multiregional resource to support mechanistic studies in human tissue and animal models.

## Highlights

• Hemisphere-resolved cortical transcriptomic atlas across 13 Brodmann areas
• Hemisphere-dependent regional expression patterns revealed across human cortex
• rs62060948 links MYC binding to right BA44 WNT3; MYC knockdown lowers WNT3
• Left striatal Wnt3 alters mouse vocalizations, locomotion, and MBP expression

## Graphical abstract

**Figure:**
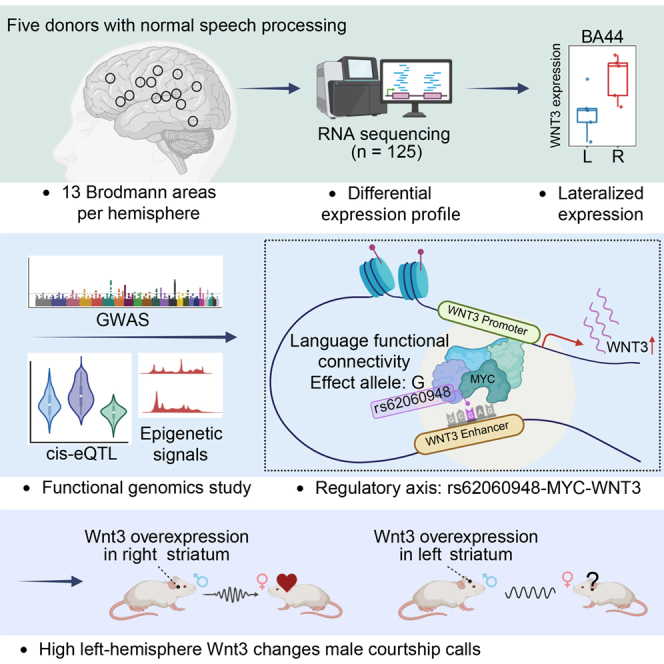

## Introduction

Human speech is a unique vocal communication system.^1^ Its evolution enabled the complex societies and cumulative cultures that define our species.^2^ This capacity is not a single trait, but rests on several key biological foundations. The human vocal tract underwent significant anatomical changes to produce a wide range of sounds.^3^ Concurrently, our brains evolved specialized neural circuits for vocal learning, a rare ability among mammals.^4^ These developments were supported by genetic innovations.^5^ Understanding how these anatomical, neural, and genetic systems became integrated remains a central question in human evolution.

A major milestone in the genetic understanding of human speech was the identification of *FOXP2*, a transcription factor located on chromosome 7q31, as the first gene linked to speech and language development.^6^ Subsequent studies revealed additional genetic factors within the *FOXP2*/7q31 locus that regulate common language impairments, with *FOXP2* truncations emerging as novel causes of developmental speech and language deficits.^7^ Beyond *FOXP2*, the growing field of human genetics has illuminated the polygenic contributions to language-related disorders. Genome-wide association studies (GWASs), an unbiased approach to studying complex traits, have identified numerous single-nucleotide polymorphism (SNP) loci associated with language-related disorders, including stuttering^8^ and dyslexia.^9^ These discoveries underscore the intricate genetic architecture of human speech processing.

Despite these advancements, the causative mechanisms linking genetic variants to speech production remain unclear. Integrating GWAS findings with expression quantitative trait loci (eQTL) analyses offers a powerful strategy to bridge this knowledge gap. This approach enables the identification of functional variants and their regulatory roles in brain-related traits, paving the way for deeper insights into the genetic basis of speech processing.^10^

Human speech processing contains two aspects of production and perception, primarily involving the functional neuroanatomy of the left hemisphere, encompassing critical regions such as Broca’s area, Wernicke’s area, Geschwind’s area, the ventral sensory-motor cortex (vSMC), and the primary auditory cortex (pAC). While the left hemisphere is typically dominant for human speech,^11^ the right hemisphere also contributes to certain linguistic functions, indicating a nuanced interplay between hemispheres.^12^ It is noted that vocalization is the prerequisite and foundation for human speech production. While prior studies reported interhemispheric expression differences and lateralization in human language cortex,^13^^,^^14^ a hemisphere-resolved resource across many speech processing-related regions with mirrored sampling and genetic integration has been lacking.

In this study, we profiled 125 RNA sequencing (RNA-seq) samples from 13 speech-related Brodmann areas (BAs) bilaterally in five adult donors. The atlas revealed region-specific expression with hemisphere-dependent differences, including higher *WNT3* in the right BA44. Integration of brain *cis*-eQTLs, enhancer annotations, and genome-wide association signals for speech-, language-, and reading-related traits prioritized a regulatory axis linking rs62060948 to MYC binding and *WNT3* expression, supported by MYC occupancy and knockdown in cell lines. Considering vocalization is the basis of speech, and in mice, dorsal striatum is known to be critical in vocal production. We found murine left striatal *Wnt3* overexpression (*Wnt3* OE) altered ultrasonic vocalizations (USVs), locomotion, and myelin basic protein levels, suggesting WNT3 as a candidate modulator of lateralized cortico-striatal circuits relevant to vocalization and motor control.

## Results

### Neuroanatomical organization of speech processing-related brain regions

**Figure 1:**
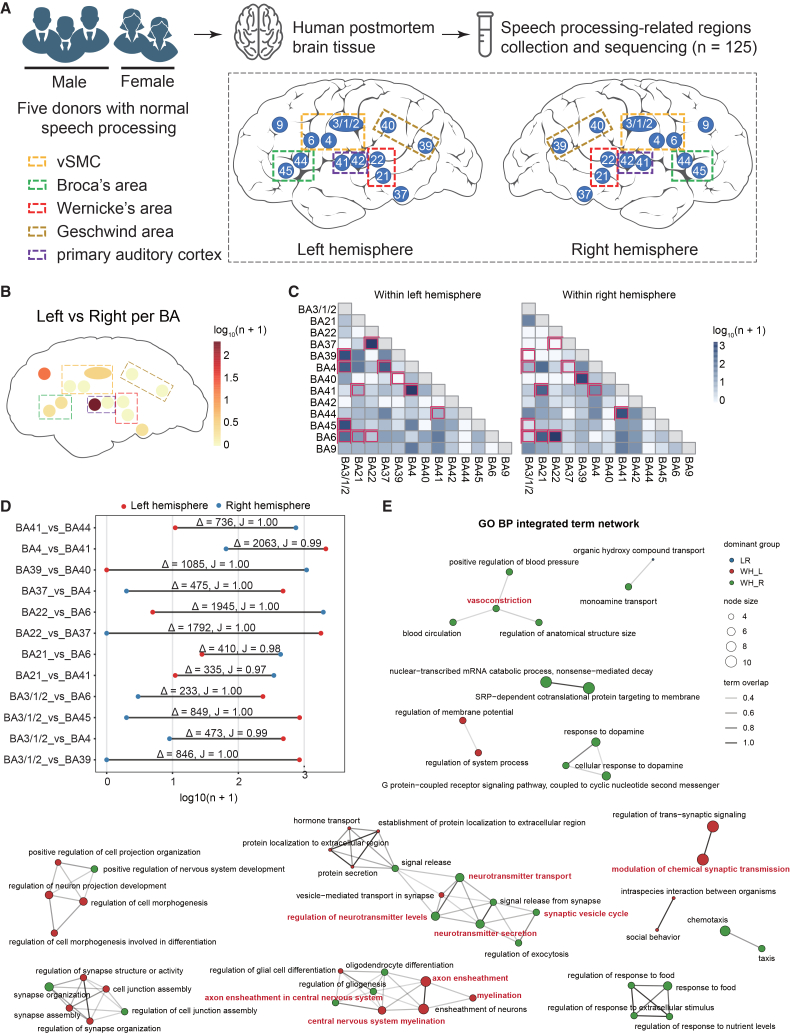
Transcriptomic profiling of speech processing-related areas in the human brain (A) Speech processing-related regions and sample collection. (B) Left-versus-right differential expression per area. Color encode the number of significant genes per BA [log_10_(*n* + 1)]. (C) WH pairwise contrasts. Heatmaps show, for the left hemisphere and for the right hemisphere separately, the numbers of significant genes for each BA-to-BA comparison [log_10_(*n* + 1)]. The 12 combinations with the greatest differences are marked in red. (D) Dumbbell plot of hemispheric differences in WH inter-areal contrasts. For each BA pair, blue and red dots represent the number of significant genes (FDR <0.05) in the left and right hemispheres, respectively. Horizontal bars connect hemisphere-specific values and are annotated with Δ, the absolute difference in significant gene counts, and *J*, the Jaccard distance between left and right gene sets. (E) GO Biological Process integrated term network. Nodes represent enriched terms drawn from three comparison families: LR, WH_L, and WH_R. Node size reflects the number of genes annotated to the term; edge thickness reflects term overlap. Terms related to synaptic transmission, neurotransmitter handling, vesicle cycling, axon and myelin biology, and vasoactive processes are highlighted.

Here, we briefly introduce the standard criteria for sample selection based on neuroanatomical organization. We analyzed a total of 125 samples, encompassing 13 speech processing-associated BAs in each hemisphere, bilaterally obtained from the brains of five donors aged 63 to 87 years (Figure 1A, Table S1). The 26 cortical BAs were collected at a 5-mm radius, and their positions were mapped to the 32k_fs_LR space according to the anatomical positions in postmortem tissue and the whole-brain Brodmann atlas.^15^ All donors were free of any personal or family history of speech processing-related psychiatric or neurological disorders. RNA-seq was performed on all samples to explore the transcriptional landscape of these regions.

We profiled key speech processing regions, as shown in Figure 1A. Targets included Broca’s area (BA44 and BA45),^16^^,^^17^ Wernicke’s area (BA22 and BA21),^18^^,^^19^^,^^20^ inferior parietal lobule including Geschwind’s area (BA39 and BA40),^21^ pAC (BA41 and BA42),^22^ and ventral sensorimotor cortex vSMC (BA6, BA4, and BA3/1/2).^23^^,^^24^^,^^25^ We also included BA37 and BA9 given their relevance to visual word processing and reading in Chinese.^26^^,^^27^ This selection defines the anatomical framework for subsequent transcriptomic analyses.

### Gene expression patterns of human speech processing-related brain regions

Transcriptomic profiling of human speech processing-related brain regions provides valuable insights into the molecular mechanisms underlying speech processing. To assess the stability of gene expression patterns across all samples, we first analyzed the total number of expressed genes and the expression levels of housekeeping genes.^28^ Despite minor variations in the total number of expressed genes being observed across brain regions and among individual donors, the consistent expression of housekeeping genes across all samples (Figure S1A) underscores the high quality and reliability of the dataset.

We constructed a hemisphere-resolved transcriptomic atlas of speech-related cortical regions by profiling five adult donors bilaterally across predefined BAs. Using donor-paired, covariate-adjusted models that accounted for sex, age, handedness, family history of language disorders, and personal history of language disorders, we performed two classes of comparisons: left versus right (LR) hemisphere within the same area, and within-hemisphere (WH) contrasts between different area pairs (Figure S1B, Tables S1 and S2). LR analyses revealed significant differential expression across multiple cortical regions, with the number of affected genes varying substantially by area. Notably, BA41 exhibited 215 significantly differentially expressed genes (DEGs) (FDR<0.05) (Figure 1B, Table S3), indicating that transcriptional lateralization is region-specific rather than uniform across the cortex. WH inter-area comparisons further demonstrated structured patterns that differed between hemispheres, as visualized by distinct footprints of significant area-to-area differences in left- and right-hemisphere heatmaps (Figure 1C). To validate the robustness of these DEGs, we incorporated disease datasets from public databases, focusing on conditions affecting normal speech processing. All expression profiles were derived from the human cortex (Figure S2A). Batch correction effectively reduced variability between our cohort and the external datasets (Figure S2B). We then conducted DEG analysis between different brain regions in our cohort and disease datasets to explore gene expression differences. Notably, 58.8% (2,261/3,844) of DEGs identified within our cohort were also detected as differentially expressed in at least one disease dataset (Figure S2C), supporting the biological relevance of these genes across independent cortical cohorts. Because the disease datasets span multiple cortical regions and conditions and were not designed to test hemispheric effects, this overlap should not be interpreted as hemisphere-specific replication.

Next, we ranked cortical area pairs by interhemispheric divergence using a dumbbell summary that compares the number of DEGs per inter-area contrast between hemispheres (Figure 1D). This metric incorporates both the absolute difference in gene counts (Δ) and the Jaccard distance (*J*) between hemisphere-specific gene sets. The largest differences were seen in pairs involving BA3/1/2. For example, BA3/1/2 versus BA39, BA4, BA45, and BA6 showed Δ values of 2063, 1945, 1792, and 1085, respectively, all with *J* values near 1.00, reflecting minimal overlap between left and right gene sets for the same contrast. Other high-ranking pairs included BA21 versus BA41 and BA21 versus BA6, which had large Δ values, and BA39 versus BA40 and BA41 versus BA44, which showed *J* values of at least 0.97. These findings indicate a model of hemisphere-dependent inter-area regulation rather than a uniform lateralized shift across regions.

To place these patterns in a functional context, we integrated enrichment across LR, within left hemisphere (WH_L), and within right hemisphere (WH_R) comparisons into a single network that highlights recurrent terms in *trans*-synaptic signaling and vesicle cycling, neurotransmitter transport and secretion, axon ensheathment and central nervous system myelination, and blood circulation with vasoactive processes (Figure 1E). The synaptic vesicle cycle and regulated exocytosis are established as core mechanisms of neurotransmission, so their prominence here is consistent with the known biology of cortical communication.^29^^,^^30^ Adult oligodendrocyte differentiation and adaptive myelination are now understood to be modulated by neuronal activity and to contribute to circuit tuning and learning, which supports the appearance of axon ensheathment and myelination terms in our integrated analysis.^31^^,^^32^ Terms related to blood circulation and vasoactive signaling align with current views of neurovascular coupling, in which neurons, astrocytes, and vascular cells coordinate cerebral blood flow to match local activity.^33^ Together, these literature-anchored themes reinforce that hemisphere- and area-dependent transcriptional differences converge on processes that support neuronal signaling, myelin biology, and neurovascular support functions within the same statistical framework used to build the atlas.

Focusing on key regions involved in speech processing, including Broca’s area, Wernicke’s area, Geschwind’s area, pAC, and vSMC, we subsequently performed hierarchical clustering and weighted gene co-expression network analysis (WGCNA) (Table S4). We selected the soft-thresholding power that best approximated a scale-free topology for network construction (Figure S3A). To examine relationships between gene modules and these regions, we visualized the topological overlap matrix as a heatmap (Figure S3B). The hierarchical clustering dendrograms revealed discrete gene co-expression modules that delineate region-specific transcriptional programs (Figure S3C). Modules are shown with unique color labels, highlighting the complexity of gene expression across these brain areas. Within the identified gene modules, the gene-gene interaction network of a prominent myelination-enriched module (module MEblack in Figure S3D) highlighted key hub genes, including PLP1 and mitochondrial genes (MT-ND1, MT-ND4, MT-ND5), which are integral to processes such as myelination and brain metabolism.^34^^,^^35^ These hub genes serve as central connectors within co-expression networks of speech-related brain regions, consistent with roles in supporting cortical circuit function. Strong co-expression connections were represented by intense red areas, indicating tightly interconnected gene networks. The relationships between specific modules and brain regions were examined, and transcript level correlations were observed (Figure S3E). For instance, the MEblack module demonstrated a significant positive correlation with BA4 (*r* = 0.23, *p* = 0.009), although no significant associations were observed with its left or right subdivisions. Conversely, the MEyellow, MEblue, and MEgrey modules exhibited significant associations with left BA37 (*r* = −0.24, *p* = 0.008; *r* = 0.23, *p* = 0.01; *r* = 0.3, p = 7e-04, respectively). Additionally, MEgrey genes correlated significantly with left BA44 (*r* = 0.23, *p* = 0.009).

These findings illustrate that hemisphere and region are associated with specific gene modules within the speech-related cortex. Given the modest sample size and lack of functional speech measurements, these associations should be viewed as an initial map of transcriptional organization rather than a comprehensive or causal description of human speech processing. Together, these analyses provide a resource for exploring the transcriptional architecture underlying human speech and language networks.

### Integration of DEGs with GWASs identified functional speech processing-associated loci

**Figure 2:**
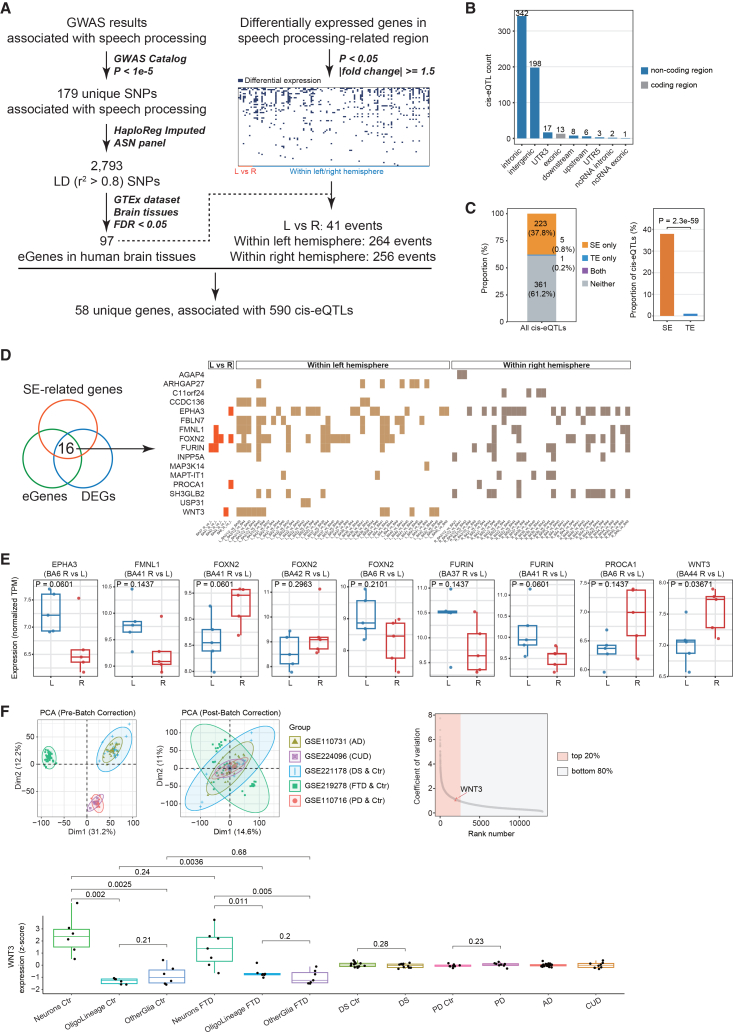
Integrating transcriptome and genome reveals candidate speech processing genes and neuronal *WNT3* (A) Combining GWAS results and nominal DEGs (*p* < 0.05 and |fold change ≥1.5|) identified hundreds of speech processing-related potentially functional loci. (B) Location distribution of 590 speech processing-related *cis*-eQTLs in genomic regions. (C) Proportion of speech processing-related *cis*-eQTL overlapping SEs versus TEs. Statistical comparison was performed using McNemar’s exact test. (D) Colocalization analysis of eGene, DEGs, and SE-related genes of the human brain identified 16 candidate genes related to speech processing. (E) Normalized gene expression (TPM) levels for the 6 brain lateralization-associated DEGs were compared across different BAs. A Wilcoxon test was used for statistical analysis. (F) Neuronal-specific expression of *WNT3* in speech processing-related contexts. Principal-component analysis (PCA) of brain transcriptomes across various neurodevelopmental and neurodegenerative conditions, shown before and after batch correction using TPM values. The adjacent ranking plot displays genes by coefficient of variation (CV), with the top 20% most variable genes highlighted in pink and the bottom 80% in green. Boxplots show the expression levels (*Z*-score normalized TPM) of *WNT3* across disease cohorts, including AD, CUD, DS, FTD, and PD. Statistical significance was assessed by *t* test.

Genetic factors have been implicated in the etiology of speech- and language-related disorders.^36^^,^^37^ To explore potential molecular links between these traits and our transcriptomic atlas, we integrated GWASs of speech, language, and reading phenotypes with nominal DEGs defined at a liberal threshold *p* < 0.05 and |fold change| ≥ 1.5 from speech processing brain regions (Figure 2A). This uncorrected threshold was used only to generate an inclusive candidate list for integrative analysis and external validation, rather than to make definitive claims about individual DEGs.

A total of 190 association results across 18 behavioral, psychological, and cognitive traits related to speech, language, or reading (for example, stuttering, and dyslexia) were compiled from the GWAS catalog^38^ (Table S5). From these, we obtained 179 unique index SNPs associated with these speech-, language-, or reading-related traits. To expand the catalog of potentially functional variants, we performed linkage disequilibrium (LD) analysis on the 179 index SNPs. LD-based imputation yielded 2,793 additional SNPs, which we intersected with the *cis*-eQTL data from the GTEx Project across 13 brain tissues. Across the 13 brain tissues, we observed *cis*-eQTL loci for WNT3 in cortical regions, subcortical regions, and cerebellum (Table S6), with cortical tissues contributing a substantial fraction of loci for the candidate eGenes (636 loci and 38 eGenes). Collapsing these loci identified 97 unique eGenes associated with speech processing. To further relate these eGenes to speech processing regions, we extended differential expression analyses beyond homologous BAs and performed pairwise comparisons among specific areas within the left hemisphere and within the right hemisphere. Intersecting all DEGs with the 97 eGenes identified 58 overlapping genes, corresponding to 41 interhemispheric differential expression events, 264 left intrahemispheric events, and 256 right ones (Table S7).

These 58 genes were significantly associated with 590 SNP genotypes across human brain tissues, most of which mapped to intergenic or intronic regions (Figure 2B). Given the regulatory potential of non-coding regions, we investigated the enhancer-like functions by interacting with key transcription factors, thus regulating the expression of target genes.^39^ Specifically, colocalization analysis of the 439 SNPs with super enhancer (SE) and typical enhancer (TE) regions revealed marked enrichment. Notably, 223 of 590 SNPs (37.8%) were uniquely located within SE regions, whereas only 5 SNPs (0.8%) mapped to TE regions (Figure 2C). These findings underscore the central role of SEs in regulating gene expression associated with speech processing.

To investigate SE regulatory activity in brain tissue, we restricted colocalization to human brain-specific SE annotations curated from public resources described in STAR Methods. We identified 16 candidate eGenes under putative SE regulation that were differentially expressed (*p* < 0.05 and |fold change| ≥ 1.5) in speech processing regions (Figure 2D). Several genes showed intrahemispheric differences, including *AGAP4* and *C11orf24* within the left hemisphere and *ARHGAP27* and *CCDC136* within the right hemisphere. Other genes exhibited interhemispheric lateralization, including *EPHA3* in BA6, *FMNL1* in BA41, *FOXN2* in BA41, BA42, and BA6, *FURIN* in BA37 and BA41, *PROCA1* in BA6, and *WNT3* in BA44. These patterns are consistent with hemispheric specialization of language-related circuitry and with lateralized gene expression in human language-related areas.^13^^,^^14^^,^^40^ To validate the differential expression signals with an emphasis on hemispheric lateralization, we applied Wilcoxon tests to normalized TPM values and retained genes that remained consistently significant in LR comparisons. Notably, *WNT3* showed significantly higher expression in right BA44 than in left BA44 (Figure 2E), a pattern that aligns with reports that the right inferior frontal gyrus including BA44 contributes to prosodic and suprasegmental aspects of speech,^41^ although this anatomical correspondence is suggestive and does not establish a direct causal role for WNT3 in speech. Given the developmental roles of WNT signaling and that thalamic WNT3 directly regulates neocortical FOXP2 translation,^42^^,^^43^ as well as UK Biobank results identifying *WNT3* among genome-wide loci for handedness and linking handedness to language-network connectivity,^44^ we prioritized *WNT3* for downstream analysis. We next examined chromatin features and transcription factors in brain tissue and relevant cell lines, aiming to clarify mechanisms regulating *WNT3* expression. Notably, *WNT3* was found to be enriched for H3K27ac (Table S8), an epigenetic marker strongly associated with active enhancer regions,^45^ suggesting its regulatory significance. Furthermore, transcription factor analysis indicated that *WNT3* regulation may involve MYC, a transcription factor with extensive roles in gene expression (see STAR Methods).

Deteriorating speech performance is widely associated with diverse neurodevelopmental, neuropsychiatric, and neurodegenerative disorders. We therefore evaluated the potential involvement of *WNT3* by analyzing its expression across datasets for Down syndrome (DS),^46^ cocaine use disorder (CUD),^47^ Alzheimer’s disease (AD),^48^ frontotemporal dementia (FTD),^49^ and Parkinson’s disease (PD),^50^ after batch correction to control for study-specific effects (Figure 2F). Among the analyzed genes, *WNT3* ranked in the top 20% for expression change across datasets, highlighting its potential involvement across these conditions. Notably, *WNT3* showed significantly higher expression in neurons than in other cell types in both control and FTD groups.

These findings collectively nominate *WNT3* as a gene of interest in circuits that support speech and related cognitive functions, particularly given its asymmetric expression in BA44 and regulation by rs62060948 and MYC. However, the available data do not establish that *WNT3* is specific to speech or that its expression differences are sufficient to cause speech phenotypes, and should instead be viewed as generating testable hypotheses for future work.

### The enhancer variant rs62060948 modulates *WNT3* expression through MYC

**Figure 3:**
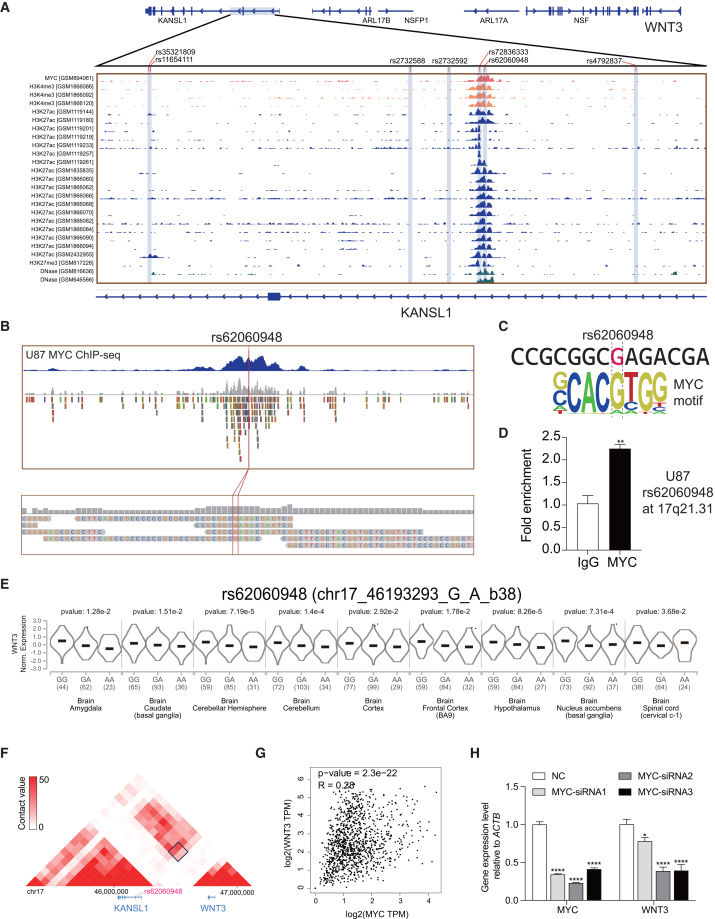
The enhancer variant rs62060948 regulates speech processing-related gene *WNT3* by influencing MYC (A) Binding of the transcription factors and the enrichment of histone modification epigenetic marker as well as chromatin accessibility on the *cis*-eQTLs of *WNT3*. (B) ChIP-seq data show that MYC can bind at rs62060948 in U87 cells. (C) rs62060948 could reside within the MYC DNA-binding motif. (D) ChIP-qPCR confirmation of MYC binding at rs62060948 region, bars represent mean ± SEM. (E) Violin plots of the association of rs62060948 genotype with the RNA expression of *WNT3* in the GTEx dataset. (F) The Hi-C dataset presented by the 3D genome Browser reveals a strong interaction (black matrix) of rs62060948 with *WNT3* promoter region in U87-MG. (G) Spearman correlation analysis of expression levels of *WNT3* and MYC in merged brain tissues dataset of GTEx. (H) Knockdown of MYC in U87 cells diminishes the mRNA levels of *WNT3*, bars represent mean ± SEM.

To investigate the regulatory mechanisms underlying *WNT3* expression, we identified speech processing-related *cis*-eQTLs for *WNT3* and integrated these with epigenomic data to pinpoint functional loci. Our analysis revealed two SNPs, rs62060948 and rs72836333, located within the *KANSL1* gene, enriched with *MYC* peaks, active histone markers (H3K27ac, H3K4me3), and chromatin accessibility marker (DNase I) (Figure 3A). These findings highlight their potential as functional enhancer variants. Notably, rs62060948 was recently identified as a lead SNP associated with speech processing,^51^ annotated as 17:44,270,659_G_A (hg19), with G as the effect allele.

To confirm its regulatory role, we analyzed MYC ChIP-seq data from U87 cells, remapped reads to the genome, and identified the G allele consistently present at rs62060948 (Figure 3B). Motif analysis revealed that rs62060948 resides within the DNA-binding motif of MYC, with the G allele enhancing predicted MYC binding affinity (CGAG) compared to the A allele, which has significantly lower binding affinity (Figure 3C). This supports the hypothesis that functional noncoding variants can modulate transcription factor DNA-binding affinity.^52^^,^^53^ To validate transcription factor binding at rs62060948, we performed ChIP followed by quantitative PCR (ChIP-qPCR) in U87 cells, confirming significant MYC enrichment at the enhancer region containing rs62060948 (Figure 3D).

Notably, the allele G to A in rs62060948 was significantly correlated with reduced *WNT3* expression in the majority of 13 types of brain tissues (9 out of 13) in a large cohort of individuals (Figure 3E). In contrast, despite its high LD with rs62060948 (ASN, LD r2 = 0.86), rs72836333 has a minor allele frequency of zero in Asian populations (1000 Genomes Project) and does not exhibit a significant association with *WNT3* expression in human brain tissues (Figure S4). The variant rs62060948 located in an intron of *KANSL1*, is approximately 570 kb upstream of *WNT3*. Analysis of public Hi-C data in U87-MG cells via the 3D Genome Browser^54^ suggests a long-range chromatin interaction between the region harboring rs62060948 and the *WNT3* promoter (Figure 3F). Moreover, a significant positive correlation between *WNT3* and *MYC* expression was observed across GTEx brain tissue datasets (Figure 3G, *R* = 0.28, *p* = 2.3e-22).

To further elucidate the regulatory role of *MYC* on *WNT3* expression, we performed siRNA-mediated knockdown experiments targeting *MYC* in U87 cells. Knockdown of *MYC* using three independent siRNAs resulted in a significant reduction in *WNT3* transcript levels (Figure 3H).

Taken together, these findings establish rs62060948 as a functional enhancer variant that modulates *WNT3* expression by influencing the chromatin binding of MYC. The evidence highlights rs62060948 as a plausible regulatory locus for *WNT3* with potential relevance to speech- and language-related genetic associations.

### Lateralized modulation of mouse vocalization and locomotion by striatal *Wnt3* OE

In humans, Broca’s area, including BA44, is related to speech production. Our study demonstrated that *WNT3* expression is significantly higher in the right BA44 compared to the left BA44. Mice do not have speech but have vocalization. Dorsal striatum is involved in vocal production in mammals.^55^ To explore potential functional consequences of altered *Wnt3* signaling in circuits relevant to vocal behavior, we manipulated *Wnt3* in mouse brain regions that participate in USV. The dorsal striatum not only integrates cortical, thalamic, and midbrain dopaminergic inputs but has also been implicated in selecting and initiating motor programs underlying orofacial movements and vocalizations.^56^^,^^57^ Besides, anterior cingulate cortex (ACC) and periaqueductal gray (PAG) are also involved in eliciting social USVs^58^ and driving brainstem motor circuits that execute vocal patterns,^59^ respectively. In addition, the auditory cortex (AC) is involved in processing auditory feedback during vocalization, and mouse AC shows modulation even before call onset.^60^

**Figure 4:**
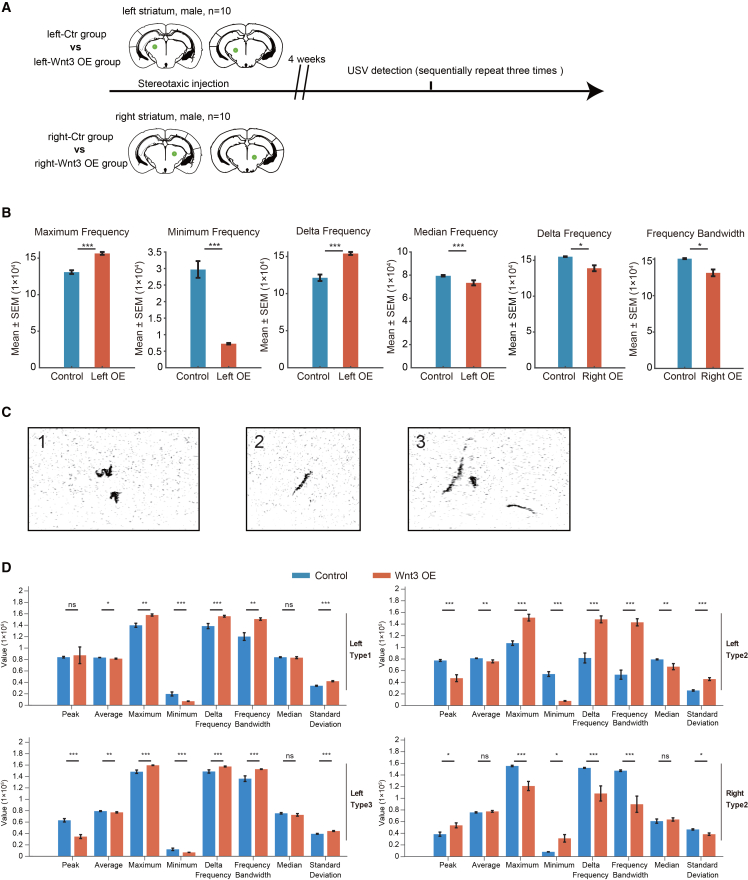
Lateralized effect of *Wnt3* OE in the left versus right striatum on USV spectral features (A) rAAV2/5-CMV-EGFP-WPRE-pA or rAAV2/5-CMV-*Wnt3*-P2A-EGFP-WPRE-pA were stereotaxically injected into the left or right striatum of 8-week-old mice (*n* = 10 male mice per group). Four experimental groups were included: left-Ctr, left-*Wnt3* OE, right-Ctr, and right-*Wnt3* OE. (B) Quantification of USV spectral parameters (two-tailed Wilcoxon test), bars represent mean ± SEM. (C) Representative spectrograms showing three classified USV syllable types based on spectral-temporal patterns (types 1–3). (D) Detailed comparison of USV spectral parameters across syllable types and hemispheric groups. Each panel shows peak/average/maximum/minimum/delta frequency, frequency bandwidth, median frequency, and SD. Bars represent mean ± SEM; ∗*p* < 0.05, ∗∗*p* < 0.01, ∗∗∗*p* < 0.001.

To check the baseline expression of *Wnt3* in those mouse brain regions, we performed RT-qPCR in left and right dorsal striatum, ACC, PAG, and AC. A generally low expression level of Wnt3 was observed with a Ct value in the range of 32–36 (Table S9). Thus, we adopted a *Wnt3* OE strategy to probe the capacity of increased *Wnt3* signaling to alter vocalization-related behavior; we conducted *Wnt3* OE in the left or right dorsal striatum of mice and assessed vocalization performance (*n* = 10 mice per group). Specifically, AAV vectors carrying full-length *Wnt3* were stereotaxically injected into the left or right dorsal striatum of 8-week-old male mice (Figure 4A). Mice were divided into four groups: left-control (left-Ctr), left-*Wnt3* OE, right-control (right-Ctr), and right-*Wnt3* OE. Four weeks post-surgery, USV test was conducted.

During male-female courtship, we compared USV patterns across the four groups. Notably, only the left *Wnt3* OE group exhibited significant alterations in USV features compared to the left-Ctr group, including a significant increase in maximum frequency (*p* = 5.8e–11) and delta frequency (*p* = 4.1e–11), and a marked decrease in minimum frequency (*p* = 1.0e–11) and medium frequency (*p* = 0.002) (Figure 4B). In contrast, the right *Wnt3* OE group showed significant reduction in delta frequency (*p* = 0.016) and frequency bandwidth (*p* = 0.011) compared to right control group, indicating the lateralized effect of *Wnt3* OE on USV spectral modulation.

We further categorized USVs into three unique syllable types based on spectral and temporal similarities (see STAR Methods, Figure 4C). Consistently, the left-*Wnt3* OE group showed significant increases in maximum frequency (*p* = 0.003, 9.5e–6, and 3.9e–5 for 3 USV types, respectively), delta frequency (*p* = 0.005, 1.3e–5, and 0.001, respectively), and frequency bandwidth (*p* = 0.001, 4.8e–6, and 2.5e–5, respectively), while it showed marked decreases in minimum frequency (*p* = 3.6e–4, 2.3e–6, and 1.5e–4, respectively) in all three USV types (Figure 4D).

**Figure 5:**
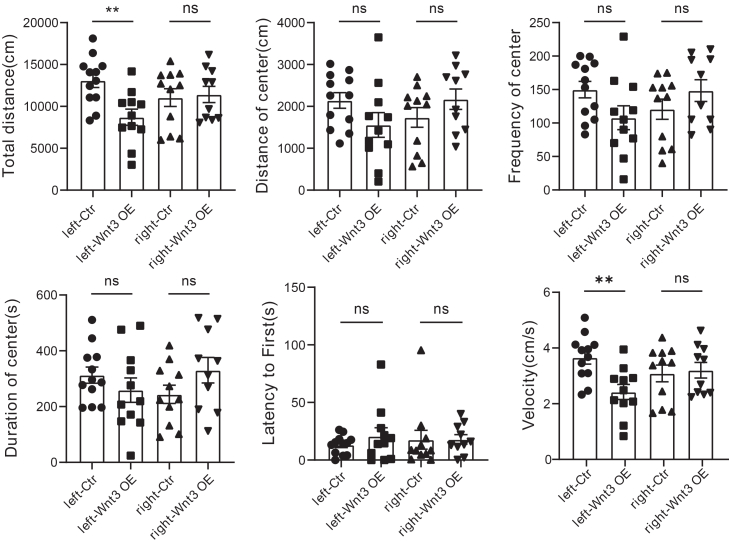
Lateralized effect of *Wnt3* OE in the left versus right striatum on locomotor ability rAAV2/5-CMV-EGFP-WPRE-pA or rAAV2/5-CMV-*Wnt3*-P2A-EGFP-WPRE-pA were stereotaxically injected into the left or right dorsal striatum of 8-week-old mice (*n* = 10 male mice per group). Four experimental groups were included: left-Ctr, left-*Wnt3* OE, right-Ctr, and right-*Wnt3* OE. In the open field test, only left-*Wnt3* OE but not right group showed reduced total distance (∗∗*p* = 0.0025) compared with controls. Bars represent mean ± SEM.

Furthermore, we performed open field test and found that left but not right *Wnt3* OE group showed reduced total distance, suggestive of its defective locomotor ability (Figure 5). The dorsal striatum is crucial for action selection and motor program initiation, including orofacial movements and vocalization-related behaviors. Together, USV and open field tests indicate *Wnt3* OE in left but not right dorsal striatum led to abnormal vocalization and reduced locomotor activity.

It was reported that during development, mouse thalamic WNT3 can promote FOXP2, a transcription factor implicated in speech and language development, while inhibiting oligodendrocyte maturation.^43^ To further understand the underlying molecular mechanism, we tested the effect of *Wnt3* OE on FOXP2 and myelination-related proteins MBP/MAG. The results showed that WNT3 protein is efficiently overexpressed in left and right dorsal striatum, FOXP2 expression is not obviously affected, and MBP shows a significant reduction in left but not right dorsal striatum (Figures S7G–S7H). Given the modest sample size, these data suggest an effect of *Wnt3* OE on myelination.

Together, these results indicate that left, but not right, dorsal striatal *Wnt3* OE can induce lateralized changes in spectral features of USVs and locomotor activity, with preliminary evidence for accompanying alterations in myelination-related protein expression.

## Discussion

Humanity has made remarkable progress in large language models, a key area of artificial intelligence. Recent studies highlight that natural language processing is central to communication between machines and humans.^61^^,^^62^ However, our understanding of how human language especially speech processing is intricately regulated at the molecular level remains limited. This study provides a hemisphere-resolved transcriptomic analysis of 13 human speech processing-related cortical BAs in both hemispheres, with the goal of mapping how gene expression varies across regions that support speech and language. By comparing differential gene expression across cortical regions, our findings provide a valuable resource for molecular studies of speech and language networks and offer initial insights into transcriptional correlates of hemispheric specialization.

Through integrated transcriptomic, genomic, and epigenomic analyses, we identified that the rs62060948-MYC-*WNT3* regulatory axis as a candidate contributor to circuits that support speech and language. Our results suggest that MYC preferentially binds to the G allele of rs62060948 within a super-enhancer region, enhancing *WNT3* expression. Intriguingly, *WNT3* showed higher expression in the right than in the left BA44, echoing the known hemispheric asymmetry of inferior frontal contributions to prosodic aspects of speech. Disruption of such expressional lateralization in mouse dorsal striatum, that is left overexpression of WNT3, led to altered vocal frequency, which is related to prosody.

The Wnt family comprises a group of secreted glycoproteins crucial for axis formation and midbrain development.^63^ Although we found a generally low expression of Wnt3 in mouse brain, others do report that knockdown of Wnt receptors such as Axin2, a negative Wnt/beta-catenin signaling regulator and indirectly affecting Wnt3 expression, can alleviate injuries on mitochondrial biogenesis and dopaminergic neurons in dorsal striatum.^64^ Another study indicated that blockage of several Wnts cause degeneration of adult mouse striatal synapse and disruption of motor behavior.^65^ In the adult brain, *WNT3* and *WNT3A* have been implicated in hippocampal neurogenesis,^66^ activity-dependent synaptic plasticity and long-term potentiation,^67^ regulation of glucose metabolism in cortical neurons,^68^ and modulation of synaptic vesicle recycling and excitatory neurotransmission in mature hippocampal circuits.^69^^,^^70^^,^^71^ These adult functions, particularly the links to activity-dependent synaptic signaling, provide a framework for considering how altered *Wnt3* signaling might influence circuits related to vocalization in our experiments.

Humans possess the unique ability of speech processing, while mice communicate through USVs, such as the isolation-induced USVs from pups or courtship-induced USVs from males to females.^72^ Our experiments provide evidence that *Wnt3* signaling can have lateralized consequences for vocal behavior in the adult mouse brain. Specifically, *Wnt3* OE in the left, but not right, dorsal striatum was associated with altered USV patterns, hypoactivity, and a reduction in the myelin marker, MBP. Although these data do not establish *Wnt3* as a dedicated speech gene, they suggest that asymmetric modulation of *Wnt3* signaling can influence left-sided circuits that contribute to vocal behavior and motor control, and associated with disrupted myelination.

Building on the striatal findings, we further observed that *Wnt3* OE in the left AC significantly perturbed USV spectral features (Figure S5) and downregulated key oligodendrocyte and metabolic genes, including Opalin and components of oxidative phosphorylation (Figure S7), without altering pre-vocal or vocal-related calcium activity (Figure S6). This pattern is compatible with a model in which *Wnt3* affects auditory feedback circuits, potentially through effects on myelination, so that degraded signal fidelity in left-hemispheric projections from AC to the secondary motor cortex (M2),^73^^,^^74^ and may contribute to abnormal motor command generation and USV spectral changes.

While our study demonstrates that left *Wnt3* OE can alter the acoustic structure of USVs, the ethological and communicative significance of these changes remains an open question. It is possible that these spectral modifications could influence how the calls are perceived. Future work, ideally conducted in collaboration with ethologists and linguists, will be highly encouraged to explore this possibility. Employing playback experiments to assess the behavioral responses to these altered vocalizations will be a critical next step in determining whether these Wnt3-driven changes carry meaningful information for mouse communication.

Taken together, our results are consistent with *Wnt3* contributing to lateralized modulation of a distributed vocal network, in which its overexpression in left-hemisphere nodes compromises both striatal control of vocalization and auditory feedback for vocal precision, potentially *via* impaired myelination. Future studies should focus on elucidating the downstream targets of *Wnt3* and its interactions with signaling networks, including MYC-regulated pathways and eQTL-associated genes. A deeper understanding of these molecular mechanisms may inform future work on the neural basis of speech and related vocal behaviors.

### Limitations of this study

This study provides insights into the lateralized role of *WNT3*; however, several aspects invite further investigation. First, the human cortical atlas is based on postmortem tissue from five donors. Although we used donor-paired designs, covariate adjustment, and multiple-testing correction to define the main left-right and WH contrasts, the modest sample size limits power to detect subtle effects and increases the risk that some detected DEGs may not replicate. The atlas should therefore be viewed as an initial resource that generates hypotheses about hemispheric and regional differences, rather than as a definitive catalog. Second, in the integrative analysis linking DEGs with GWAS loci, we intentionally used a liberal, nominal differential expression threshold (*p* < 0.05 and |fold change| ≥ 1.5) without additional multiple testing correction to assemble an inclusive candidate gene set for eQTL and enhancer colocalization. This strategy not only facilitates hypothesis generation and cross dataset comparisons but also means that the resulting candidate list is expected to contain false positives. Consequently, the implicated genes, including *WNT3*, should be interpreted as prioritized candidates rather than confirmed effectors of speech or language related traits. Third, an important future direction will be to elucidate the precise cellular mechanisms by which *WNT3*-mediated myelination defects lead to specific vocal alterations, connecting molecular changes to circuit dysfunction and behavioral output. Furthermore, establishing the direct functional relevance of the human rs62060948-MYC-*WNT3* axis, along with the ethological significance of altered vocalizations in mice, will be crucial next step in fully unraveling the molecular continuum of vocal communication.

## Resource availability

### Lead contact

Requests for resources and further information should be directed to the lead contact, Linya You (email: lyyou@fudan.edu.cn). Materials and information will be provided on reasonable request.

### Materials availability

This study did not generate new unique reagents that require deposition. AAV constructs and plasmid maps used for striatal Wnt3 manipulations will be shared by the lead contact subject to a simple material transfer agreement when required.

### Data and code availability

• Human RNA-seq data have been deposited in the Genome Sequence Archive for Human (GSA-Human) under accession number HRA006169. Publicly available datasets and resources used in this study include GEO series GSE221178, GSE224096, GSE110731, GSE219278, and GSE110716, the U87 MYC ChIP-seq dataset GSM894061, GTEx v8 brain eQTL data (GTEx Portal), and GWAS summary statistics from the NHGRI-EBI GWAS Catalog.
• Analysis code is available at GitHub: https://github.com/zxwang-cloak/Human_brain_transcriptomics
• Any additional information required to reanalyze the data reported in this paper is available from the lead contact upon request.

## Acknowledgments

Z.W. was supported by the 10.13039/100014718National Natural Science Foundation of China (#82203416). W.L. and L.Y. were supported by STI2030 - 10.13039/100020441Brain Science and Brain-Inspired Intelligence Technology Major Project (#2021ZD0201100 Task 4 #2021ZD0201104) from the 10.13039/501100002855Ministry of Science and Technology (MOST), China. G.-H.W. was supported by the 10.13039/501100012166National Key R&D Program of China (#2022YFC2703600 Task 4 #2022YFC2703604) and the 10.13039/501100009962Shanghai International Collaborative Project (#23410713300). M.Z. was supported by the 10.13039/100014718National Natural Science Foundation of China (#T2122007) and Shanghai Pilot Program for Basic Research - Fudan University 21TQ1400100 (25TQ013). X.L. was supported by Fudan's Undergraduate Research Opportunities Program (FDUROP) Xi Yuan project (254401). The computation source in this work was supported by the Medical Science Data Center at 10.13039/501100015359Shanghai Medical College of Fudan University and High-Performance Computing Platform of Suzhou Institute of Systems Medicine, Chinese Academy of Medical Sciences & Peking Union Medical College.

## Author contributions

Z.W. performed genomic analyses. Y.C. performed experimental validations. Y.F. conducted USV analyses. G.Z. performed initial behavioral tests. W.W. helped with USV and calcium detection setting. L.L. performed ChIP experiments. X.L. and Y.M. helped with animal housing. J.Y., T.W., and M.L. supported data analysis. L.J. assisted in data collection. R.G.S., B.H., and M.C. provided critical comments. L.C. and S.-B.L. helped with experimental design. J.W. provided helpful comments on speech processing. Z.W., Y.C., and Y.F. prepared the initial draft of the manuscript. Z.W., M.Z., W.L., G.-H.W., and L.Y. supervised the study and finalized the manuscript.

## Declaration of interests

The authors declare no conflicts of interest.

## STAR★Methods

### Key resources table

**Table:** 

REAGENT or RESOURCE	SOURCE	IDENTIFIER
**Antibodies**		

Rabbit anti WNT3	Proteintech	Cat# 28156-1-AP RRID: AB_3669649
Rabbit anti FOXP2	Proteintech	Cat# 20529-1-AP RRID: AB_10695756
Rabbit anti MAG	Proteintech	Cat# 14386-1-AP RRID: AB_2878051
Rabbit anti MBP	Oasis	Cat# OB-PRB130-02 RRID: AB_3717395
Rabbit anti GAPDH	Proteintech	Cat# 10494-1-AP RRID: AB_2263076
HRP Goat anti Mouse IgG H L	Proteintech	Cat# SA00001-1 RRID: AB_2722565
HRP Goat anti Rabbit IgG H L	Proteintech	Cat# SA00001-2 RRID: AB_2722564

**Biological samples**		

Human postmortem brain tissue	Fudan brain bank	Ethics 2019C025

**Chemicals and recombinant proteins**		

Trizol	Thermo Fisher	Cat# 15596026
EZ 10 DNAaway RNA Mini preps Kit	Sangon Biotech	Cat# B618133-0050
SYBR qPCR Master Mix	Vazyme	Cat# Q711-02
HiScript III qRT SuperMix	Vazyme	Cat# R323-01

**Deposited data**		

Human brain RNA-seq	GSA Human	HRA006169 https://bigd.big.ac.cn/gsa-human/browse/HRA006169

**Experimental models: organisms/strains**		

C57BL/6J	SiPeiFu	https://spfbiotech.com/

**Experimental models: cell lines**		

U87 MG	Vendor to be confirmed	Cat# HTB-14 RRID:CVCL_C9F7

**Oligonucleotides**		

ChIP-qPCR primers rs62060948 region	This study	Forward AGCCCCACAAAATGGGCGCAG Reverse TGCGGAGCAAGGCCGGGAAA

**Recombinant DNA**		

rAAV2/5-CMV-Wnt3-P2A-EGFP WPRE-hGH-pA	This study	titer 5.0E+12 vg per mL production
rAAV2/5-CMV-EGFP-WPRE-hGH-pA	This study	titer 5.0E+12 vg per mL production

**Software and algorithms**		

R	R Project	v4.0.5 https://www.r-project.org
DESeq2	Bioconductor	v 1.30.1 https://bioconductor.org/packages/DESeq2
limma	Bioconductor	v3.46.0 https://bioconductor.org/packages/limma
edgeR	Bioconductor	v3.32.1 https://bioconductor.org/packages/edgeR
sva ComBat-Seq	Bioconductor	v3.38.0 https://bioconductor.org/packages/sva
SOAPnuke	BGI	v2.1.9 https://github.com/BGI-flexlab/SOAPnuke
FastQC	Babraham	v0.11.9 https://www.bioinformatics.babraham.ac.uk/projects/fastqc
HISAT2	Kim Lab	v2.2.1 http://daehwankimlab.github.io/hisat2
Bowtie2	Johns Hopkins	v2.2.5 http://bowtie-bio.sourceforge.net
RSEM	Dewey Lab	https://deweylab.github.io/RSEM
DAVID	NCI	https://davidbioinformatics.nih.gov
DeepSqueak	Coffey Lab	https://github.com/DrCoffey/DeepSqueak
MATLAB	MathWorks	https://www.mathworks.com/products/matlab.html
Avisoft Recorder	Avisoft	https://www.avisoft.com
IGV	Broad Institute	https://igv.org
3D Genome Browser	Zhang Lab	http://3dgenome.org
JASPAR	JASPAR	2022 https://jaspar.genereg.net
CistromeDB Toolkit	Liu Lab	http://cistrome.org/db
Fiji ImageJ	NIH	https://imagej.net/software/fiji
GraphPad Prism	GraphPad	v8.0 https://www.graphpad.com/scientific-software/prism
WGCNA	CRAN	v1.73 https://CRAN.R-project.org/package=WGCNA
GEPIA2	Peking University	http://gepia2.cancer-pku.cn
HaploReg	Broad Institute	v4.2 https://pubs.broadinstitute.org/mammals/haploreg/haploreg.php
FANTOM5	RIKEN	https://fantom.gsc.riken.jp
SEdb	Zhang Lab	http://www.licpathway.net:8081/sedb/

### Experimental model and study participant details

#### Human postmortem brain tissue

Human postmortem cortical tissue was obtained from the Fudan branch of the National Health and Disease Human Brain Tissue Resource Center (Body Donation Station in Fudan University; Shanghai Red Cross Society) under ethical approval (2019C025). Five adult donors (63–87 years old; sex: 3 male/2 female) were included. Where available, donor metadata included age, sex, and handedness; information on ancestry and gender identity was provided in Table S1. Donors were free of any reported personal or family history of speech-processing-related psychiatric or neurological disorders. Thirteen Brodmann areas (BA22, BA21, BA37, BA39, BA40, BA41, BA42, BA44, BA45, BA9, BA6, BA4, and BA3/1/2) were dissected from left and right hemispheres (125 total samples). Samples were not randomized; hemisphere (left vs right) and cortical area defined the comparison groups, and donors served as matched blocks for paired analyses. Sex was included as a covariate in differential expression models; due to the limited number of donors, the study was not powered to test sex-by-hemisphere interactions, which is a limitation.

#### Animals

C57BL/6J wild-type male mice were purchased from SiPeiFu (China). Mice (8-week-old, n = 20) were under specific pathogen-free (SPF) conditions with a 12 h light/12 h dark cycle at 25°C and 50% ± 10% relative humidity, with *ad libitum* access to food and water. All animal procedures were approved by the Animal Care and Use Committee in the School of Basic Medical Sciences of Shanghai Medical College, Fudan University (approval number: 20230301-148). Male mice were used for courtship ultrasonic vocalization assays; therefore, sex-specific effects were not evaluated in mice and conclusions may not generalize to females.

#### Cell lines

U87-MG cells (RRID: CVCL_C9F7) were obtained from Cat# HTB-14 and maintained as described in METHOD DETAILS. Cell line authentication was performed by service provider. Cells were confirmed to be free of mycoplasma contamination by PCR testing (primer-F: GGGAGCAAACAGGATTAGATACCCT, primer-R: TGCACCATCTGTCACTCTGTTAACCTC).

### Method details

#### Human sample collection

Human postmortem brain tissue from five donors were obtained from Fudan branch of National Health and Disease Human Brain Tissue Resource Center, the Body Donation Station in Fudan University (Shanghai Red Cross Society), under ethical approval 2019C025. Language-related regions including BAs 22, 21, 37, 39, 40, 41, 42, 44, 45, 9, 6, 4, and 3/1/2 were dissected from left and right hemispheres by an experienced neuroanatomy lecturer.

#### Human RNA-seq library generation

Donors’ brains were dissected to collect specific BAs as indicated. The samples were put into cold DMEM and immediately transferred to the lab. After removing meninges and blood vessels, each sample was immediately put into a tube with 0.7 mL of Trizol and stored at -80°C. Total RNA was extracted using Trizol method. The RNA quality and integrity were assessed by Agilent 2100 Bioanalyzer with RNA6000 Nano kit. Only RNA with an RNA integrity number (RIN) value of more than 6.5 was processed for library preparation using the oligo (T) enrichment method, which was sequenced with paired-end 100bp and generated 8G raw data per sample.

#### Human RNA-seq processing

Raw data were filtered to remove reads with low quality, adapter contamination, and unknown high base by SOAPnuke.^75^ Clean reads were examined for data quality by FastQC and aligned to human GRCh37/hg19 using Hisat.^76^ Ribosomal RNA was removed by comparing it with the human rRNA database from NCBI using Bowtie.^77^ Count, TPM and FPKM were calculated by the RSEM package.^78^ The count matrix was then corrected for batch effect by ComBat-Seq^79^ and re-normalized to obtain TPM. DEGs between paired BAs were analyzed by DESeq2,^80^ visualized by volcano plot or heatmap, and GO analysis was performed in the DAVID platform.^81^ A paired t-test was used to compare the gene of interest using the ggpubr v0.4.0 package in R v4.0.5.

#### Probabilistic inference of handedness from multi region laterality

We inferred handedness from cortex transcriptomes using a leave one donor out design across predefined speech and motor regions of interest. In each fold we fit right versus left hemisphere contrasts within region using voom precision weights, limma linear modeling with empirical Bayes moderation, and TMM library scaling. Donor was treated as a blocking factor and within donor correlation was estimated with “duplicateCorrelation”. Moderated t statistics from the training donors defined a signed weight vector. For the held-out donor we formed region level laterality scores by projecting right minus left log expression differences onto this vector and then averaged region scores into language and motor indices. A simple Bayesian mapping converted the language index to a posterior probability of right handedness using literature informed priors. Implementation used R with limma and edgeR.

#### Donor paired differential expression for the lateralized atlas

All left right and within hemisphere Brodmann area contrasts were analyzed with a paired design at the donor level. For each contrast we restricted to donors contributing both conditions and required at least two donors. For every contrast we applied subset level expression filtering and normalization before model fitting. Genes were retained if the total count across the subset was at least 10 and if counts were greater than 0 in at least 3 samples. Size factors were estimated within the subset. Models included a donor blocking term and the relevant condition term, hemisphere for left versus right within the same area and BA for area versus area within the same hemisphere. Covariates at the sample level sex, age, handedness, family history of language disorders, and language disorders were included only when they varied within the set of donors used for that contrast to avoid collinearity with the donor term. Numeric covariates were median imputed and standardized and categorical covariates were mode imputed; covariates that were all missing or constant were dropped. Wald tests from DESeq2 were used with Cook distance outlier filtering disabled. Factor levels were set to ensure R versus L for hemisphere and BA1 versus BA2 for within hemisphere contrasts. Multiple testing was controlled at several levels. We reported the Benjamini and Hochberg false discovery rate within each contrast, a global false discovery rate pooling all P values across genes and contrasts, a family wise control across Brodmann areas for the left right analyses, and a family wise control across six predefined functional modules Broca, Wernicke, Geschwind, primary auditory, ventral sensorimotor, and other. Pairs that spanned different modules were labeled mixed and were not adjusted at the module level. Unless stated otherwise all atlas figures were produced at FDR less than 0.05 after covariate adjustment; the lenient threshold mentioned previously applied only to an independent replication step and not to atlas construction.

#### WGCNA and network construction

Gene expression data were analyzed using the WGCNA^82^ package in R. Data preprocessing involved the removal of missing values from the TPM expression matrix, Genes with high coefficients of variation, calculated as the standard deviation divided by the mean expression level, were retained for network construction. Quality control measures included hierarchical clustering to identify and exclude outlier samples. Soft-thresholding power was determined by identifying the point at which the scale-free topology fit index reached a plateau, selecting a power of 6. The network was then constructed using a signed network type to preserve the direction of gene relationships, with a minimum module size set at 30 genes to ensure robust module detection. The resultant modules were visualized in a dendrogram and annotated with colors corresponding to different gene modules. For each module, hub genes were identified by calculating the connectivity of each gene within the module and selecting the gene with the highest connectivity. Module eigengenes, representing the first principal component of each module, were correlated with external trait data, which recorded the origin of samples, specifically indicating which hemisphere and language-related region of the brain each sample was from. The correlations and their p-values were calculated using Pearson’s method, and results were displayed in a heatmap to identify significant associations.

#### Speech processing-related genetic loci collection

All associations (v1.0.2) from the NHGRI-EBI GWAS Catalog^38^ on 29/11/2022 were downloaded to search the reported genetic loci related to human speech processing. The associations with the traits related to speech processing, including stuttering, dyslexia, and other phenotypes related to language disorders were collected (Table S1). For the SNPs with noncanonical rsID in associations, their genome position in the linked literature was checked one by one. Considering that the SNPs in the GWAS Catalog have been mapped to GRCh38/hg38, we used the UCSC Liftover tool to convert the genome position of noncanonical SNP from hg19/hg18 to hg38 genome builds. For LD analysis, we applied standard post imputation quality control to language related GWAS SNPs, selected sentinel variants with P < 1e-5, and performed LD expansion in HaploReg^83^ using an ancestry matched panel ASN, retaining high LD proxies with r^2^ > 0.8 within a symmetric window to yield 2,793 SNPs; enrichment of this SNP set for brain cis-eQTLs was then assessed using the GTEx v8 database.^84^

#### eQTL analysis and gene expression correlation

Paired SNP-gene association was queried in GTEx eQTL Dashboard using brain tissues. Gene-gene expression correlations were queried in GEPIA2^85^ (http://gepia2.cancer-pku.cn) using merged all kinds of types of GTEx brain tissues.

#### Public RNA-seq collection and batch correction

We systematically retrieved bulk RNA-seq data from the GEO database to identify datasets associated with diseases potentially contributing to human language disorders. Initially, we conducted a search using the query “(((human brain, cortex, disorders, RNA-seq) NOT scRNA-seq) NOT mouse)” to focus on studies related to human cortex disorders, excluding single-cell RNA-seq data and non-human datasets. Following the search, we manually reviewed the entries to confirm that the selected datasets were derived from human cortex samples and were relevant to language-related functions. The verified datasets, provided in raw count format, were then processed for quality control and normalization, preparing them for subsequent analysis.

The count matrices from multiple datasets were merged, retaining only those genes that were expressed across all datasets. We then used the home-made scripts to convert the count values to TPM values. The limma^86^ package was employed for batch correction of the TPM values, and PCA was performed to assess the effectiveness of the batch correction.

#### Epigenetic data collection and processing

SE and TE region distribution were obtained from the SEdb^87^ and FANTOM5^88^ databases, respectively. For colocalization analysis with eGene and DEGs, SE regions of human brain tissue in the SEdb database were collected and annotated to genes using the Perl script “annotatePeaks.pl” in HOMER.^89^

Transcription factors or chromatin regulators that could bind in the SNP interval were scanned using Toolkit in CistromeDB.^90^ Factors that could regulate the genes of interest in brain tissue or cell lines were searched and collected with the setting of a half-decay distance to the transcription start site within 10 kb.

U87 MYC ChIP-seq raw data (GSM894061) were downloaded from the GEO dataset. Adapters were removed using TrimGalore v0.6.7 (RRID: SCR_011847), and clean data were mapped to the reference genome (hg38) using Bowtie2^77^ with default parameters. Downloaded bigwig files and aligned bam files were visualized in IGV. MYC frequency matrix in homo sapiens was downloaded from the JASPAR CORE database.^91^ U87-MG Hi-C data is from Xu et al.^92^ and the heatmap picture was generated from a 3D Genome Browser^54^ with 80kb resolution.

#### ChIP and ChIP-qPCR

U87-MG cells were cross-linked with 1% formaldehyde at room temperature for 10 min, followed by the addition of 0.125M glycine solution to stop the reaction. Cells pellets were collected, washed with cold PBS and resuspended in hypotonic lysis buffer (10 mM KCl, 20 mM Tris-HCl pH 8.0, 10% Glycerol, 2 mM DTT, protease inhibitor cocktail (Bimake)) and gently rotated 30-50 min to isolate nuclei. The nuclei were washed with cold PBS and resuspended in SDS lysis buffer (50 mM Tris-HCl pH 8.0, 10 mM EDTA, 0.5% SDS and protease inhibitor cocktail). Nuclear extracts were sonicated to generate chromatin fragment with an average size of ∼250bp using a M220 Focused-ultrasonicator (Covaris). For immunoprecipitation, 70 μl of Dynabeads Protein G (Invitrogen) was precleared with blocking buffer (0.5% BSA in IP buffer) and added to each sample, followed by incubation with 8 μg antibody against c-Myc (18583S, CST) or control IgG (normal rabbit IgG, 30000-0-AP, Proteintech) at 4°C for 12 hours. The supernatant was then removed. For chromatin binding, 300 μg soluble chromatin in IP buffer (20 mM Tris–HCl pH8.0, with 2 mM EDTA, 150 mM NaCl, 1%Triton X-100, and Protease inhibitor cocktail) was added to the bead-antibody complex and incubated for at least 12 hours. The supernatant was then removed and the bead-DNA–protein complex was washed six times with RIPA washing buffer (50 mM HEPES pH 7.6, 1 mM EDTA, 0.7% sodium deoxycholate, 1% NP-40, 0.5 M LiCl). Subsequently, the DNA–protein complex was eluted in DNA extraction buffer (10 mM Tris–HCl pH 8.0, 1 mM EDTA, and 1% SDS), followed by overnight treatment with Proteinase K and RNase A at 65 °C to reverse the crosslinks of protein-DNA interactions. Finally, DNA was purified with MinElute PCR purification kits (QIAGEN) and target DNA fragments were analyzed by quantitative real-time PCR assay. The primer sequences of ChIP-qPCR were as follow: F:AGCCCCACAAAATGGGCGCAG, R: TGCGGAGCAAGGCCGGGAAA.

#### Cell culture and RNA interference

U87-MG cells were cultured in DMEM containing 10% fetal bovine serum, 100 units/mL penicillin and 100μg/mL streptomycin at 37°C in a humidified incubator with 5% CO_2_. The synthesized siRNAs were transfected into U87-MG with Lipofectamine™ RNAiMAX Transfection Reagent (Invitrogen), and cells grown in 6-well plates were transfected with 25 pmol RNA oligonucleotides according to the manufacturer’s instructions. The siRNAs targeting human MYC were synthesized by GenePharma. Cells were harvested for subsequent experiments 48h after transfection. The efficiency of siRNAs was evaluated by RT-qPCR.

#### RT-qPCR analysis

Total RNA was extracted using EZ-10 DNAaway RNA Mini-preps Kit (Sangon Biotech). The complementary DNA was synthesized from purified RNA by 5x HiScript III qRT SuperMix (Vazyme) according to the manufacturer’s instructions. RT-qPCR was performed on a LightCycler 480 real-time PCR detection system (Roche) using SYBR qPCR Master Mix (Vazyme). The cycle threshold (Ct) values were analyzed using the 2^-ΔΔCt^ method, and the final results were presented as relative expression fold change.

#### Mouse stereotactic surgery

The animal experiments conducted in this study were approved by the Animal Care and Use Committee in the School of Basic Medical Sciences of Shanghai Medical College, Fudan University (approval number: 20230301-148). The mice were intraperitoneally anaesthetized with Avertin (M2920, Nanjing Aibei Biotechnology) and placed in a stereotactic head frame (Model 68001, RWD Life Science).

For dorsal striatum injections, rAAV2/5-CMV-EGFP-WPRE-hGH polyA or rAAV2/5-CMV-*Wnt3*-P2A-EGFP-WPRE-hGH polyA were stereotaxically injected into left or right dorsal striatum of 8-week-old mice (n=10 male mice per group). Four groups of mice (left-Ctr, left-*Wnt3* OE, right-Ctr and right-*Wnt3* OE) were included. Two sites were injected per cortex with 100 nL of AAV virus (5.00E+12 vector genome (vg)/ml). The coordinates for the injections into left dorsal striatum are AP +0.8 mm, ML -1.5 mm, DV -2.4 mm and AP +0.8 mm, ML -1.5 mm, DV -2.7 mm. The coordinates for the injections into right dorsal striatum are AP +0.8 mm, ML +1.5 mm, DV -2.4 mm and AP +0.8 mm, ML +1.5 mm, DV -2.7 mm.

To allow diffusion of the virus, the injection pipette remained immobile for 5 min before withdrawal. Subsequent experiments were performed four weeks after surgery.

#### Western blot

Mouse dorsal striatum tissue was lysed in RIPA buffer and incubated for 20 min on ice. Samples were cleared by centrifugation at 12,000 rpm for 15 min, sonicated and protein concentration was measured by BCA kit (Beyotime, P0012). Lysates were mixed with 5X SDS-PAGE sample loading buffer and boiled at 95°C for 5 min. Cell lysates were loaded onto 10% polyacrylamide gels. The proteins were then transferred to 0.45 μm PVDF membranes. Membranes were blocked with 3% BSA for 1 hr at room temperature and incubated with primary antibodies including rabbit anti-WNT3 (1:600, Proteintech, 28156-1-AP), rabbit anti-FOXP2 (1:600, Proteintech, 20529-1-AP), rabbit anti-MAG (1:2500, Proteintech, 14386-1-AP), rabbit anti-MBP (1:2500, Oasis, OB-PRB130-02) and rabbit anti-GAPDH (1:10,000, Proteintech, 10494-1-AP) in 3% BSA overnight at 4°C. Subsequently, the membranes were washed 3 times in TBST and incubated with HRP-conjugated Goat Anti-Mouse IgG(H + L) (1:5000, Proteintech, SA00001-1) and HRP-conjugated Goat Anti-Rabbit IgG(H + L) (1:5000, Proteintech, SA00001-2) for 1 hr at room temperature, followed by 3 washes. The antigen–antibody complexes were detected by ECL. Quantification of Western blots was performed using Fiji/ImageJ software.

#### USV recording

Male-female social encounter was used to elicit and record USVs with 3 main stages, Habituation, Interaction and Removal. In the Habituation step, male mouse was introduced to the testing chamber for 5 min to be familiar with a novel environment. In the Interaction step, a female mouse was introduced into the testing chamber, and the male and female were allowed to interact for up to 2 min, or until the male mouse mounted the female. If mounting occurred, the female was immediately removed. If mounting did not occur, the female was removed after 2 min. In the final Removal step, immediately after mounting, male mouse was left in the testing chamber for 2 min and USVs were recorded.

The testing chamber was placed inside a larger sound attenuating chamber. An ultrasonic microphone (UltraSoundGate CM16/CMPA, Avisoft Bioacoustics) with suspended 2 inches above the top of the testing chamber. The acoustic signal from the microphone was digitized at 375 kHz, 16 bits (UltraSoundGate 416H, Avisoft Bioacoustics) and saved as a wav file (Avisoft-recorder, Avisoft Bioacoustics).

#### USV detection

USVs emitted by male mice were detected using the DeepSqueak framework,^93^ which integrates three pre-trained neural network models: Long Rat Detector YOLO R1, Mouse Detector YOLO R2, and Rat Detector YOLO R1. These models were employed in combination to identify USVs within audio recordings, providing precise annotations of start times and durations for each detected vocalization.

Preprocessing steps were implemented to ensure high-quality signal analysis. Frequencies outside the 10–150 kHz range were filtered to remove irrelevant noise, and low-intensity signals were excluded based on power spectral density thresholds. These steps enhanced the signal-to-noise ratio while preserving relevant acoustic components of the vocalizations. After preprocessing, a comprehensive suite of acoustic features was extracted, including Mel-frequency cepstral coefficients, duration, peak frequency, average frequency, maximum frequency, minimum frequency, frequency difference between start and end points, bandwidth, median frequency, and the standard deviation of frequency. These features quantitatively characterized the USVs, providing a robust foundation for contour analysis, classification, and statistical evaluation.

#### Ultrasonic audio data preprocessing

Ultrasonic spectrograms were generated using a 512 FFT length with 50% frame overlap, and bandpass filtered to collect sounds between 25 kHz and 110 kHz from the original soundwave signals. The power spectrum of the filtered soundwave signals was calculated, and signals with power levels exceeding 1.5 times the average power were selected as an intensity threshold to remove background noise.

#### USVs identification and feature extraction

For each USV segment, the duration and the proportion of the maximum frequency (spectral purity) were calculated. Segments with a duration exceeding 5 ms and spectral purity above 25% were extracted and considered as USVs. Extracted characteristics for each USV segment included: Duration, Peak Frequency, Average Frequency, Maximum Frequency, Minimum Frequency, Bandwidth, Start Frequency, End Frequency, Median Frequency, Frequency Standard Deviation (StdDevFrequency), Pause Duration (the time between the current USV and the previous one), Pause-To-USV Duration Ratio, Jitter, and Shimmer.

#### Contour analysis and USV classification

Contour analysis and USV classification were performed using DeepSqueak’s Contour Detection and Unsupervised Syllable Clustering modules. The contour of each USV was constructed by identifying the frequency corresponding to the maximum amplitude at each time point in the spectrogram. This approach yielded a detailed temporal representation of the spectral characteristics of each vocalization.

Using these contour data, USVs were classified into distinct categories via the Unsupervised Syllable Clustering module, which employs the k-means clustering algorithm. The analysis used default parameters, including a maximum of 100 clusters and three repetitions of the clustering process. This procedure grouped USVs based on their spectral and temporal similarities, resulting in the classification of 813 male mouse USVs into 10 unique syllable types. The output included instance syllables representing each cluster and the trained model. These results established a robust framework for investigating group differences in vocalization patterns and associated acoustic features.

#### Statistical analyses of USVs

All analyses were conducted in MATLAB. Statistical analyses were conducted to assess differences in USV characteristics between experimental and control groups. First, the mean acoustic feature values for each mouse, including peak frequency, average frequency, bandwidth, and six additional parameters, were calculated and compared between the following pairs: left control vs. left high, left control vs. left low, right control vs. right high, and right control vs. right low. Statistical tests were selected based on data distribution. Second, the relative proportions of each syllable type within a mouse’s USVs were computed as a 1×10 vector, representing the fraction of USVs assigned to each syllable type. These proportions were compared across the same group pairs. Finally, syllable-level differences were examined by comparing the acoustic features of individual syllable types between the left control and left high groups. For each of the 10 syllable types, mean feature values were computed and statistically tested. For analysis of feature differences, barplots were generated for each feature in each experimental group. A Mann-Whitney U test was performed to assess feature differences across four pairs of experimental conditions: left-Ctr vs. left-shWnt3, left-Ctr vs. left-Wnt3 OE, right-Ctr vs. right-shWnt3, right-Ctr vs. right-Wnt3 OE.

### Quantification and statistical analysis

Statistical analyses were performed using R (v4.0.5), MATLAB (MathWorks), and GraphPad Prism (v8.0). Exact n, what n represents (e.g., number of human donors, cortical samples, or animals), the statistical tests used, and measures of center and dispersion are reported in the figure legends, Results, and/or the indicated tables. Data are presented as mean ± SEM. In figures, statistical significance is indicated as follows: ∗p < 0.05, ∗∗p < 0.01, ∗∗∗p < 0.001.

#### Human transcriptomic analyses

For differential expression analyses of human RNA-seq data, donor-paired designs were implemented in DESeq2 (v1.30.1) with donor as a blocking factor. Wald tests were used for significance testing. Multiple testing correction was performed using the Benjamini-Hochberg procedure to control the false discovery rate (FDR) as described in METHOD DETAILS. Where gene-level comparisons were performed on normalized expression values, paired nonparametric tests were used as appropriate.

#### Integrative genetic and epigenomic analyses

Enrichment/overlap analyses used the tests specified in the corresponding figure legends (e.g., McNemar’s exact test for enhancer category comparisons). Correlation analyses used Spearman correlation unless otherwise stated.

#### Cell-based assays

For RT-qPCR and ChIP-qPCR experiments, comparisons between two conditions were performed using two-tailed tests (parametric or nonparametric depending on distribution). The number of independent biological replicates and the statistical tests are reported in the corresponding figure legends.

#### Mouse behavioral and ultrasonic vocalization analyses

Male mice were assigned to four groups (left-Ctr, left-Wnt3 OE, right-Ctr, right-Wnt3 OE; n=10 mice per group). USV analyses were performed in MATLAB as described in METHOD DETAILS. Planned pairwise comparisons were conducted between each overexpression group and its matched control using two-tailed Mann-Whitney U tests (Wilcoxon rank-sum tests), unless otherwise stated. Data are presented as mean ± SEM unless otherwise indicated. All analyses were performed blinded to group identity when feasible.

### Additional resources

Not applicable/This study is not a clinical trial.

## Supplemental information

Document S1. Figures S1–S7

Table S1. Characteristics of the samples involved in this study

Table S2. Handedness scores derived from transcriptome data

Table S3. Summary of differentially expressed genes for left versus right hemisphere and for inter-regional contrasts within the same hemisphere after multivariable adjustment, with FDR less than 0.05

Table S4. Summary of WGCNA modules and key results

Table S5. Speech-related GWAS information curated from the GWAS Catalog

Table S6. Colocalization of 2,793 imputed SNPs with *cis*-eQTLs in GTEx brain tissues

Table S7. eGenes validated as differentially expressed in this dataset, with P less than 0.05 and absolute fold change at least 1.5

Table S8. Epigenetic datasets for WNT3 in brain tissues or neural cell lines from CistromeDB

Table S9. RT-qPCR measurements in the left and right striatum, anterior cingulate cortex ACC, periaqueductal gray PAG, and auditory cortex AC of mice

Table S10. The acoustic features of every type of USV syllables compared between left control and left Wnt3 OE groups
