# Supplementary material for: Transcriptomics and functional genomics implicate WNT3 in hemispheric lateralization of speech production
Source: iScience. 2026 Jan 14;29(2):114692. doi: 10.1016/j.isci.2026.114692 (PMC12876320; doi:10.1016/j.isci.2026.114692)
Supplement: Document S1. Figures S1–S7 [file mmc1.pdf]

iScience, Volume *29*

Supplemental information

Transcriptomics and functional genomics implicate WNT3 in hemispheric lateralization of speech production

Zixian Wang, Yanxi Chen, Yongqi Feng, Gaoyu Zu, Wenxu Wang, Limiao Liang, Xinyu Liu, Yuhang Mei, Jiaxuan Yang, Tian Wang, Mengqi Liu, Linxi Jiang, Russell G. Snell, Blake Highet, Maurice Curtis, Liang Chen, Shi-Bin Li, Jinsong Wu, Menghan Zhang, Wensheng Li, Gong-Hong Wei, and Linya You

Supplemental Figures and Legends

Figure S1

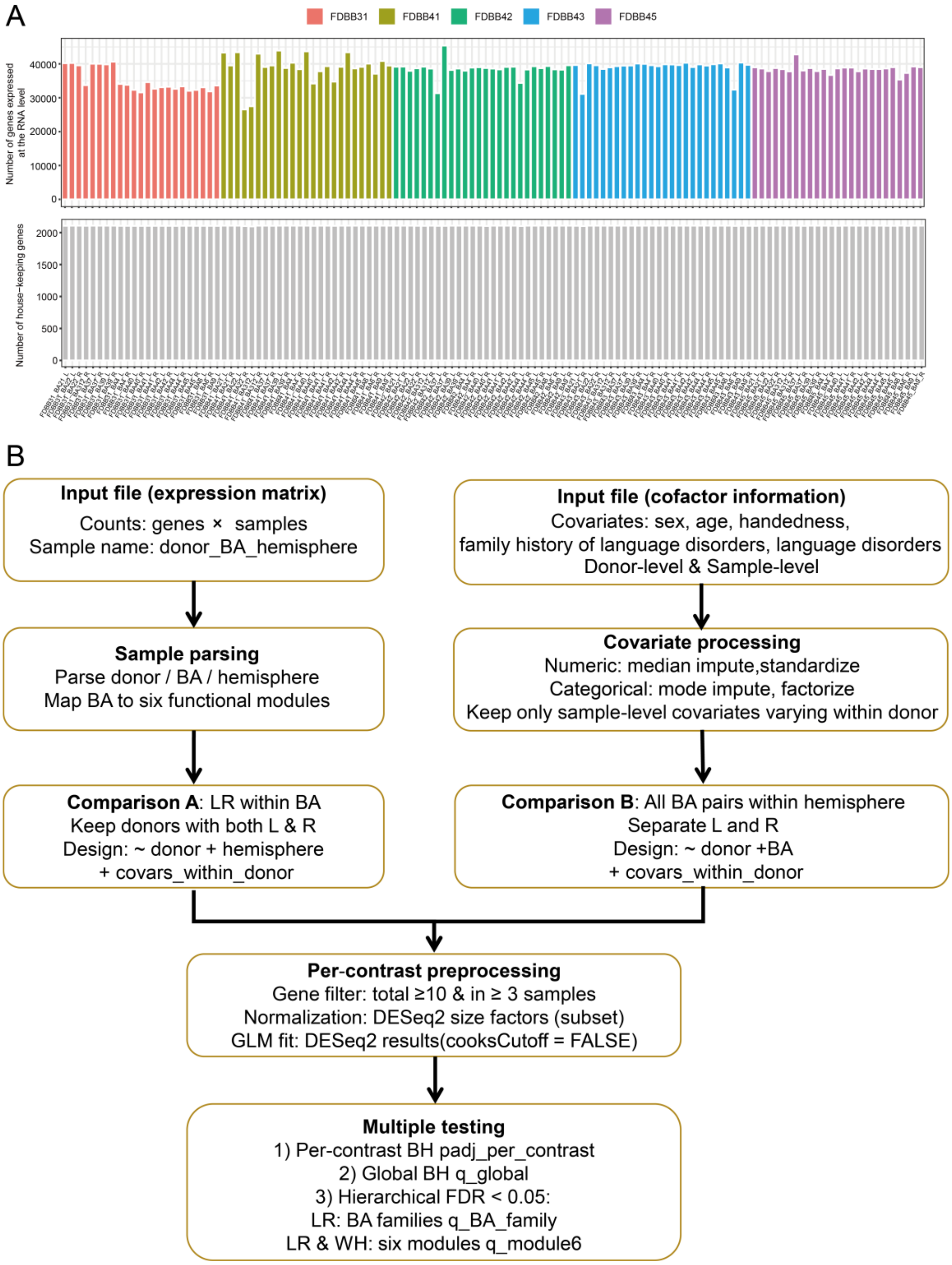

Figure S1. Regional expression and modeling strategy for the brain transcriptomic atlas, Related to Figure 1. (A) Distribution of expressed genes across brain regions. The total number of genes (top) and housekeeping genes (bottom) detected in each

region are shown. **(B)** Statistical framework for constructing the brain transcriptomic atlas. Analyses are paired by donor; only sample-level covariates that vary within donor (sex, age, handedness, family history of language disorders, language disorders) enter the model, whereas donor-level covariates are collinear with the donor term and excluded. Left–right contrasts are modeled as donor + hemisphere (+ covariates) and within-hemisphere area contrasts as donor + area (+ covariates). Differential expression is tested using Wald statistics with Benjamini–Hochberg FDR < 0.05 to define significant results.

Figure S2

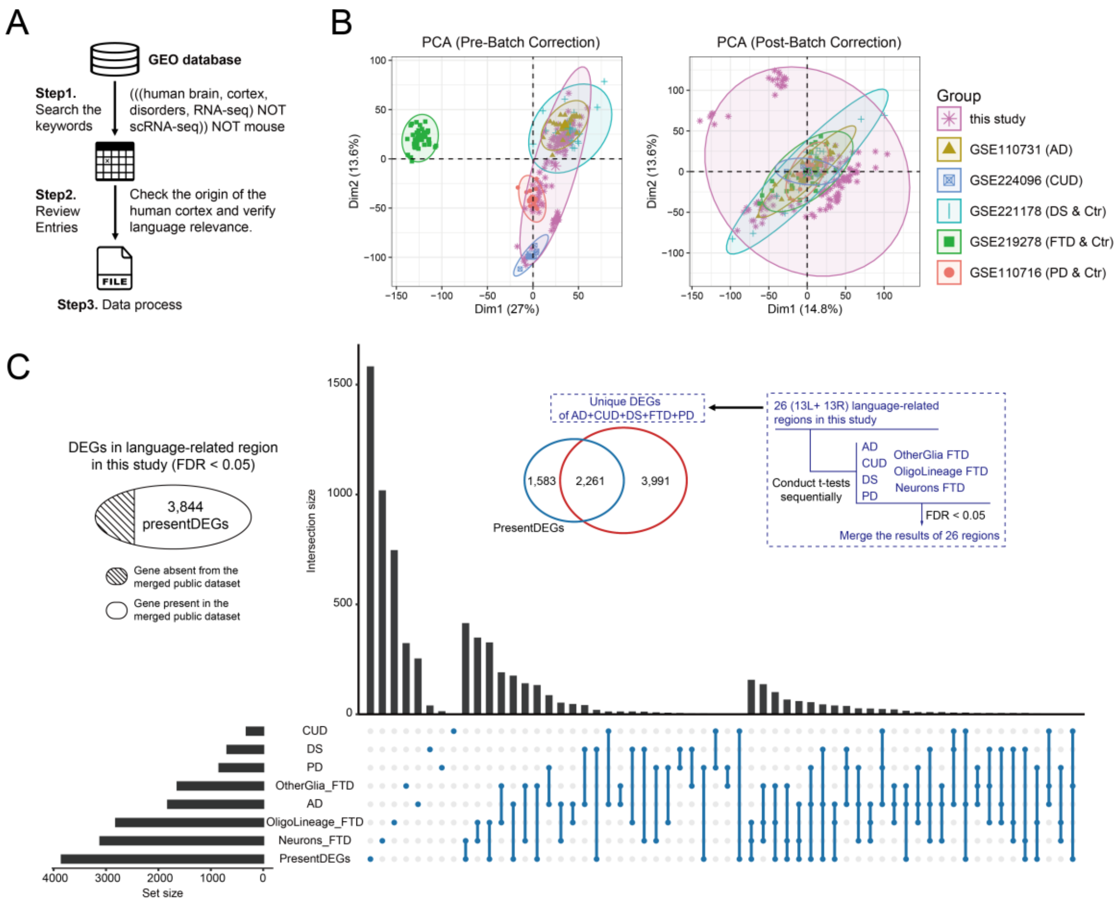

Figure S2. Validation of DEGs identified in this study using combined public data, Related to Figure 2. (A) Flowchart of the process for collecting public data. **(B)** PCA plots comparing gene expression data from this study and public datasets before and after batch correction. The left panel shows data before batch correction, and the right panel shows data after batch correction. Batch correction was performed using TPM

values converted from counts. **(C)** Validation of the reliability of DEGs (FDR < 0.05) identified under relaxed criteria in this study.

Figure S3

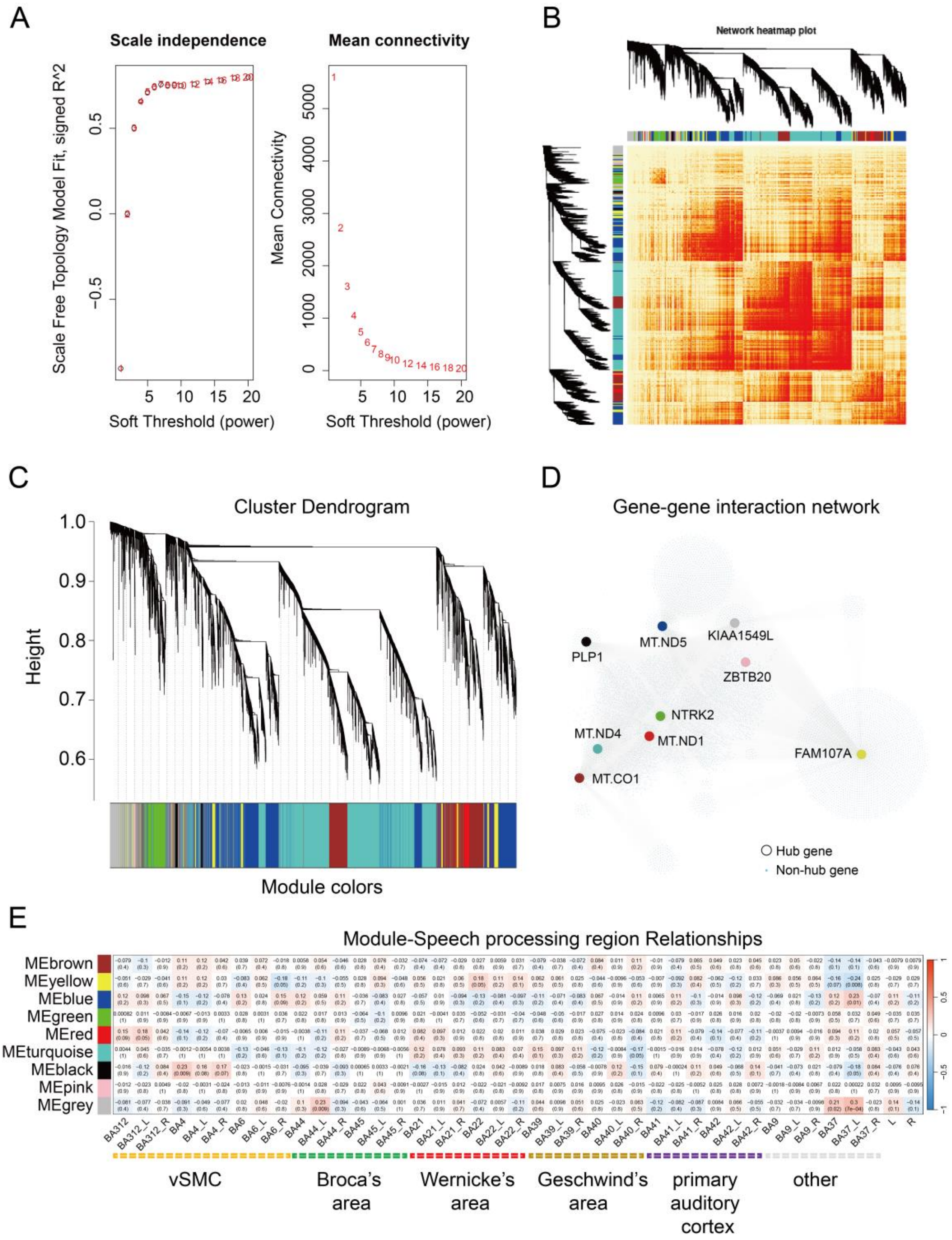

Figure S3. WGCNA of RNA-seq data from human speech processing-related regions, Related to Figure 2. (A) Scale-free topology fit index (left) and mean

connectivity (right) for various soft-thresholding powers. **(B)** Topological overlap matrix (TOM) heatmap showing the strength of gene co-expression. Rows and columns represent genes, with red intensity indicating stronger connections. Gene dendrograms and module color bars are shown along the margins. **(C)** Cluster dendrogram of expression levels of all samples. Hierarchical clustering dendrogram of genes based on topological overlap. The module colors below the dendrogram represent different gene modules identified by WGCNA. **(D)** Gene-Gene interaction network. Visualization of the gene-gene interaction network within one of the modules. Hub genes, identified as the most highly connected genes within the module, are indicated by larger nodes, while non-hub genes are shown as smaller nodes. **(E)** Module-speech processing region relationships. Heatmap showing the Pearson correlation between module eigengenes and specific speech processing-related regions. Each cell represents the correlation coefficient (r) and the corresponding p-value (in parentheses).

Figure S4

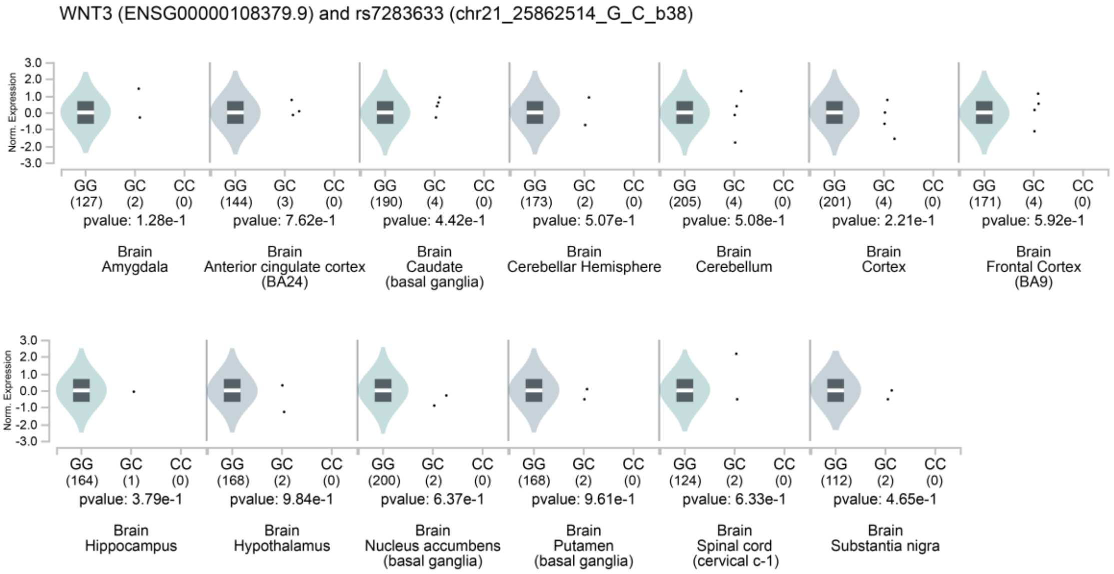

Figure S4. Violin plots of the association of rs72836333 genotype with the RNA expression of WNT3, Related to Figure 3. The number in parentheses following each genotype indicates the sample size. Data were obtained from the GTEx Portal (https://www.gtexportal.org/home/).

Figure S5

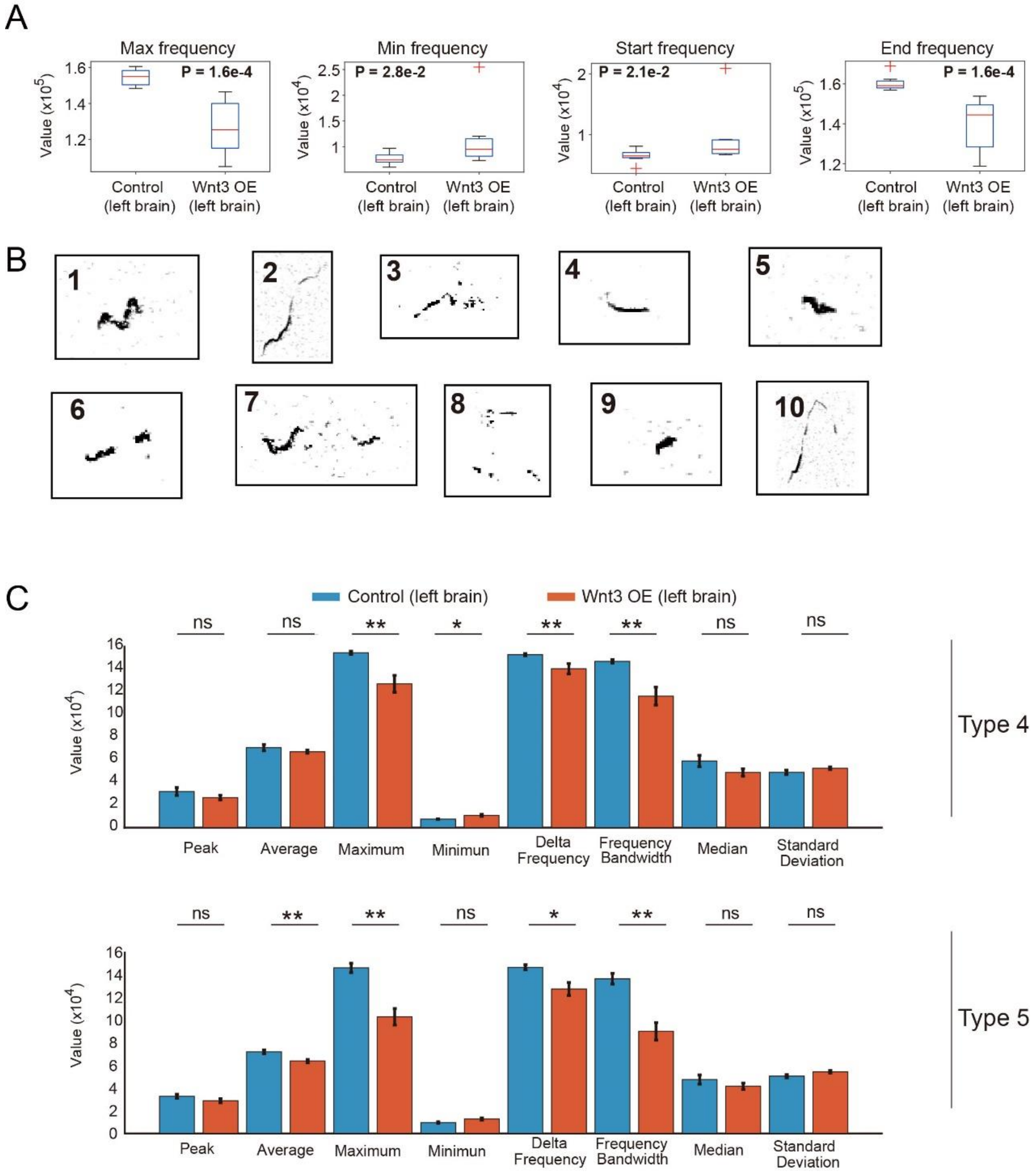

Figure S5. Acoustic alterations in ultrasonic vocalizations induced by Wnt3 overexpression in the left auditory cortex, Related to Figure 4. Recombinant adeno-associated viruses (rAAV2/5-CMV-EGFP-WPRE-pA or rAAV2/5-CMV-Wnt3-P2A-EGFP-WPRE-pA) were stereotaxically injected into the left or right auditory cortex of 8-week-old mice (n=8 male mice per group). Four experimental groups were established: left-Ctr, left-Wnt3 OE, right-Ctr, and right-Wnt3 OE. Behavioral assessments were conducted four weeks post-surgery. **(A)** USV detection was performed for male mice. Acoustic feature analysis comparing the left-Ctr and left-

Wnt3 OE groups. The left-Wnt3 OE group exhibited significantly higher minimum frequency, whereas maximum frequency, and frequency difference between start and end (Δ frequency) were reduced. **(B)** Representative USV syllables. Ten distinct syllable types were classified using an unsupervised clustering method and illustrated based on their spectral and temporal features. **(C)** Quantitative analysis of Type 4 (upper panel) and Type 5 (lower panel) USV syllables between left Ctr and left Wnt3 OE groups. $p<0.05$ *, $p<0.01$ **.

Figure S6

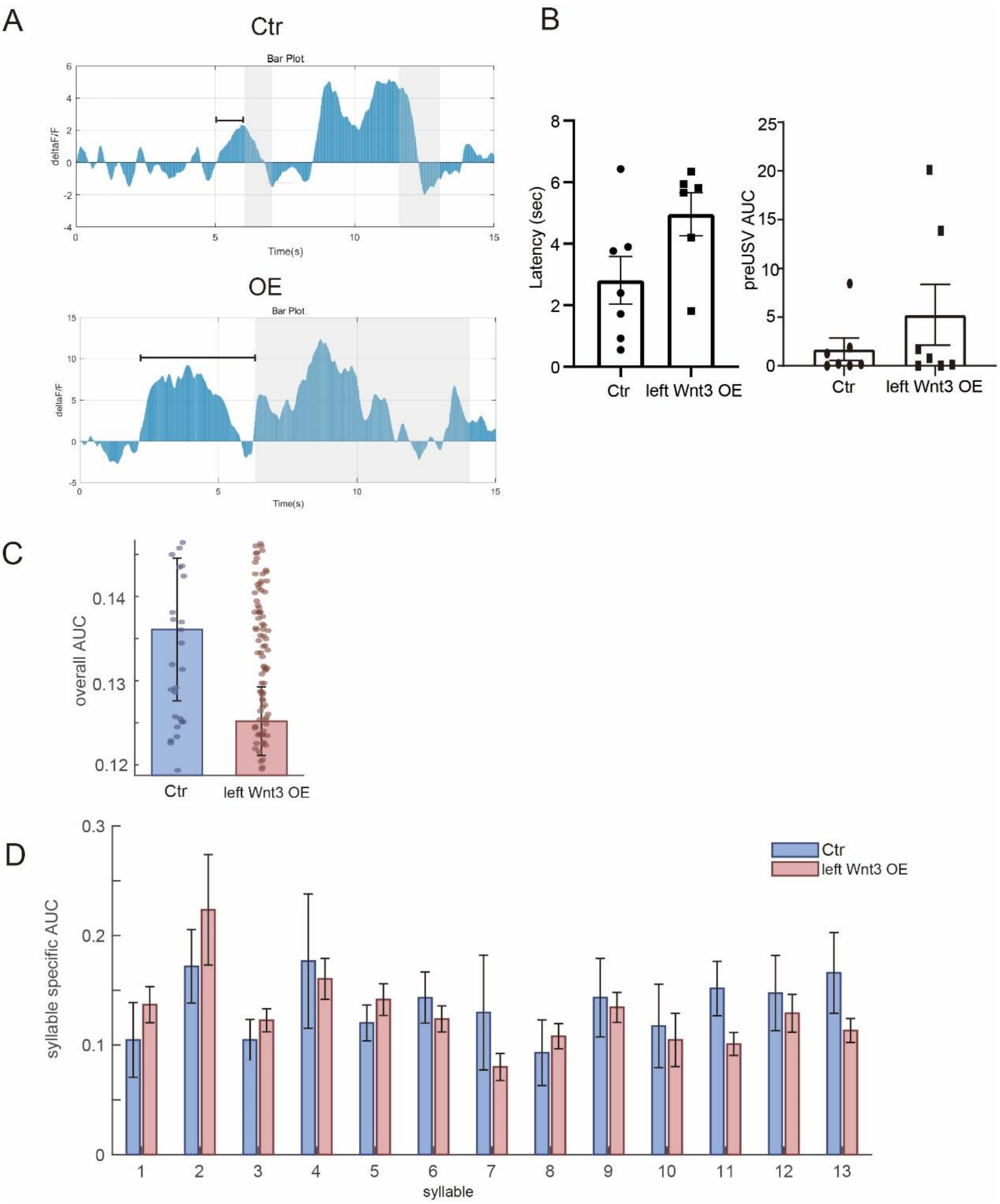

Figure S6. Calcium responses before and during USVs after Wnt3 overexpression in the left auditory cortex, Related to Figure 4. (A) ΔF/F dynamics of calcium signals recorded before and during ultrasonic vocalizations in left Ctr and left Wnt3 OE groups. The gray shading indicates the duration of USV signals, while the black line denotes the time interval between the onset of the first USV and the preceding calcium signal peak. **(B)** Left plot: comparison of the time interval between left Ctr and left Wnt3 OE

groups ($p = 0.0669$, unpaired t-test). Right plot: comparison of the AUC of calcium peaks occurring immediately before vocal onset (Wilcoxon rank-sum, $p > 0.05$). **(C)** Syllable clustering yielded 13 acoustic categories. The overall ΔF/F AUC between the left-Ctr and left-Wnt3 OE groups by pooling all syllables was compared (Wilcoxon rank-sum, $p > 0.05$). **(D)** Each of the 13 syllable categories was examined individually, and none showed a significant difference between groups (Wilcoxon rank-sum, $p > 0.05$).

Figure S7

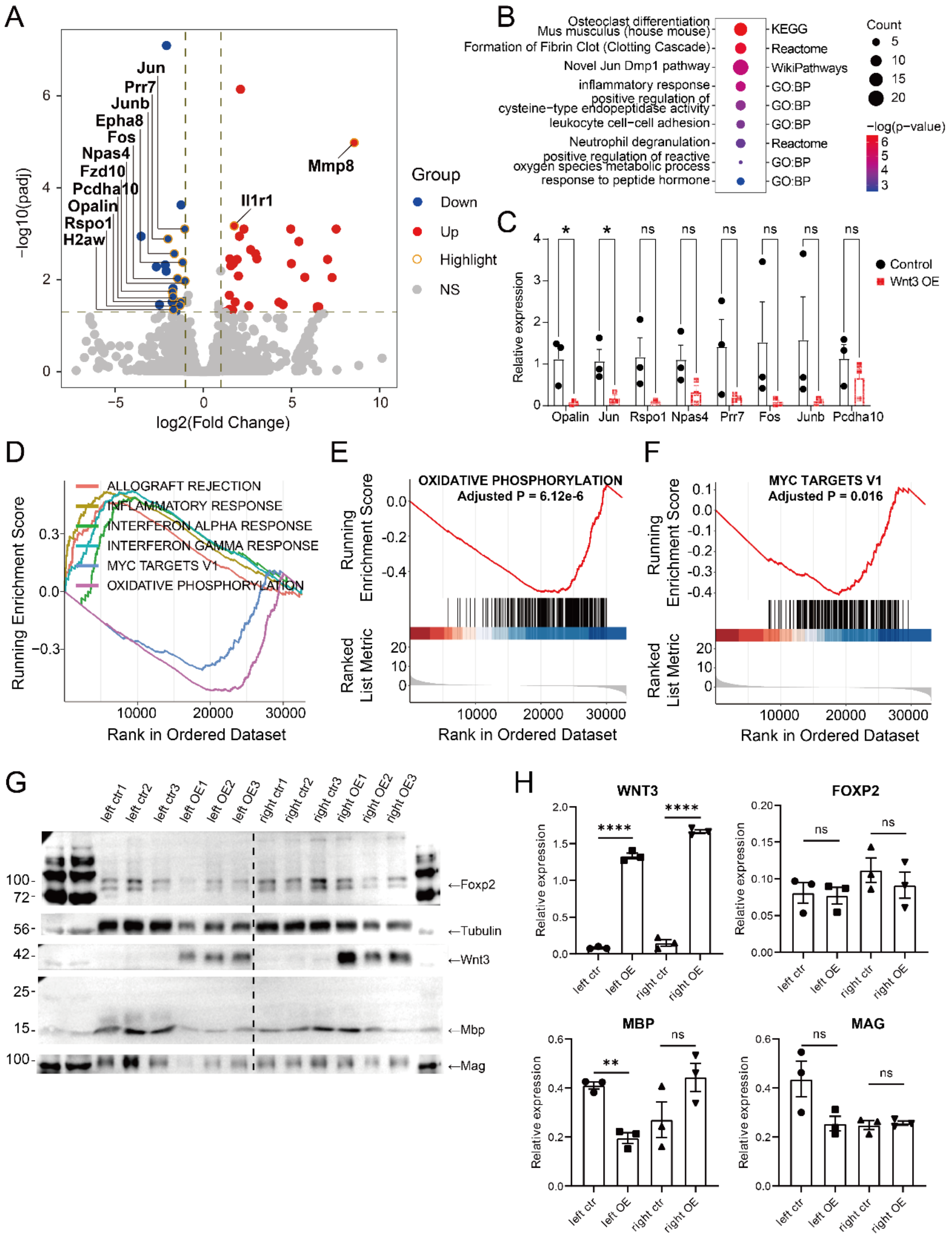

Figure S7. Effects of Wnt3 overexpression in the left auditory cortex on myelination, Related to Figure 5. (A) Volcano plot showing DEGs in the left auditory cortex of male mice with Wnt3 overexpression compared to controls. **(B)** RT-qPCR validation of key DEG mRNA levels in the left auditory cortex of Wnt3-overexpressing versus control male mice (n=3 pairs). **(C)** Pathway analysis of DEGs in the left auditory

cortex of Wnt3-overexpressing male mice, highlighting enriched biological pathways. **(D-F)** GSEA plots for significant Hallmark pathways in the left auditory cortex of Wnt3-overexpressing male mice, including all significantly enriched pathways (D), OXIDATIVE PHOSPHORYLATION (E), and MYC TARGETS V1 (F). **(G)** Representative Western blot images of striatal protein expression (n = 3 per group). **(H)** Quantitative analysis of protein expression for FOXP2, WNT3, MBP, and MAG. WNT3 was efficiently overexpressed in both left and right OE groups ($p < 0.0001$), while only left OE group showed reduced MBP expression ($p = 0.0012$). Unpaired t-test was performed.

Supplementary Schemes and Results

Mouse stereotactic surgery

The animal experiments conducted in this study were approved by the Animal Care and Use Committee in the School of Basic Medical Sciences of Shanghai Medical College, Fudan University (approval number: 20230301-148). The mice were intraperitoneally anaesthetized with Avertin (M2920, Nanjing Aibei Biotechnology) and placed in a stereotactic head frame (Model 68001, RWD Life Science).

For auditory cortex injections, the same AAV viruses as above were injected into left or right auditory cortex of 8-week-old mice (n=8 male mice per group). Four groups of mice (left-Ctr, left-Wnt3 OE, right-Ctr and right-Wnt3 OE) were included.Two sites were injected per cortex with 100 nL of AAV virus (5.00E+12 vector genome (vg)/ml). The coordinates for the injections into left auditory cortex are AP -2.46 mm, ML -4.00 mm, DV -0.50 mm and AP -2.54 mm, ML -4.20 mm, DV -0.75 mm. The coordinates for the injections into right auditory cortex are AP -2.46 mm, ML +4.00 mm, DV -0.50 mm and AP -2.54 mm, ML +4.20 mm, DV -0.75 mm.

For USV and calcium signal detection experiment, rAAV2/5-CMV-3XFlag-WPRE-hGH pA or rAAV2/5-CMV-Wnt3-3XFlag-WPRE-hGH pA with rAAV2/9-hysn-Gcamp6s-WPRE-hGH pA were stereotaxically injected into left auditory cortex of 8-week-old mice (n=8 male mice per group). Group left-Ctr and left-Wnt3 OE were included. Two sites were injected per cortex with 100 nL of AAV virus and AAV vectors

carrying genes encoding GCamp6s (5.00E+12 vector genome (vg)/ml). The coordinates are the same as above. A glass fiber (200 µm diameter, 0.37 NA, Inper) was implanted with the tip at the injection site for later GCamp6s signal acquisition.

To allow diffusion of the virus, the injection pipette remained immobile for 5 min before withdrawal. Subsequent experiments were performed four weeks after surgery.

Mouse RNA-seq processing

RNA-seq data quality was assessed using FastQC (v 0.11.9) (www.bioinformatics.babraham.ac.uk/projects/fastqc/). Low-quality reads were trimmed and adapters were removed using Trim Galore (v 0.6.7) (https://www.bioinformatics.babraham.ac.uk/projects/trim_galore/). Clean data were mapped to the mouse reference genome (mm39) using STAR (v 2.7.9a) (1) and the aligned BAM files were sorted using SAMtools (v 1.13) (2). Reads for genes were counted and the normalized expression TPM value were calculated using StringTie (v 2.2.1) (3). DESeq2 (4) package was used to generate the DEGs (|Fold Change| > 2, FDR < 0.05) and the pathway enrichment of the DEGs were performed using Metascape (5) (https://metascape.org).

Gene Set Enrichment Analysis (GSEA)

GSEA was conducted using the R package clusterProfiler (6). The analysis focused on the Hallmark gene sets obtained from MSigDB (https://www.gsea-msigdb.org/gsea/msigdb/index.jsp) (7), with Enrichment Scores (ES) computed to identify overrepresented gene sets at the extremities of the ranked gene list. Statistical significance of the ES was determined via permutation tests, where class labels were randomized to establish a null distribution. Based on these results, Nominal P-values and False Discovery Rates (FDR) were calculated. Gene sets with significant enrichment (FDR < 0.05) were identified as potentially relevant to the biological context of our study.

Results

Alterations in ultrasonic vocalizations and myelination gene expression induced by Wnt3 overexpression in the left but not right mouse auditory cortex

To investigate whether Wnt3 also influences regions involved in processing speech feedback, we conducted Wnt3 overexpression (Wnt3 OE) in the left and right auditory cortices of mice, and assessed USV signals (n = 8 male mice per group). Mice were divided into four groups as mentioned above (**Figure 4A**). During male-female interaction, we compared USV patterns across the four groups. Notably, only the left Wnt3 OE group exhibited significant alterations in USV characteristics compared to the left-Ctr group. These alterations included a significant increase in minimum frequency ($p = 0.028$) and start frequency ($p = 0.021$), while having a marked decrease in maximum frequency ($p = 0.00016$) and end frequency ($p = 0.00016$) (**Figure S5A**).

We further categorized USVs into 10 unique syllable types (**see Methods, Figure S5B**). For Type 4 USVs, the left-Wnt3 OE group showed significant decreases in maximum frequency ($p = 0.002$), delta frequency ($p = 0.001$), and frequency bandwidth ($p = 0.004$). In contrast, minimum frequency showed a significant increase ($p = 0.03$). For Type 5 USVs, significant reductions were observed in the left-Wnt3 OE group for average frequency ($p = 0.007$), maximum frequency ($p = 0.002$), delta frequency ($p = 0.02$), and frequency bandwidth ($p = 0.003$) (**Figure S5C**). Apart from this, we conducted comparisons for other eight Types (**Table S10**). Similarly, for Type 3 and Type 7 USVs, minimum frequency showed a significant increase and frequency bandwidth showed a significant reduction. Notably, for Type 2 USVs, no comparisons could be made due to that this type only exists in the left OE group but not in the left-Ctr group.

The mouse auditory cortex shows asymmetric activity when mice hear and perceive USVs. To test the possibility that a set of neurons in the left auditory cortex may get activated and be prepared for the onset of USVs, we performed USV recording and calcium signal detection simultaneously. Specifically, AAV vectors carrying full-length Wnt3 without EGFP and calcium indicator GCamp6s with EGFP were stereotaxically injected into the left auditory cortex of 8-week-old male mice (n = 8-10 mice per group). Mice were divided into two groups: left-control (left-Ctr) and left-Wnt3 OE. Four weeks' post-surgery, USV signal and calcium signal were detected simultaneously. First, we looked into the latency, the time interval from the start time

of the nearest calcium peak to the vocal onset (as indicated in **Figure S6A**), and found no significant difference between left Ctr and left OE groups (**Figure S6B** left plot). Similarly, we compared area under curve (AUC) of calcium peaks that occurred immediately before vocal onset (pre-vocal peaks), and no significant difference was found between groups (Wilcoxon rank-sum, $p > 0.05$) (**Figure S6B** right plot). This suggested that under normal status, some neurons in the left auditory cortex get prepared and are activated before the onset of vocalization, and Wnt3 overexpression does not significantly alter this phenomenon. Then, we quantified calcium responses using AUC of ΔF/F signals for each detected USV syllable (see Methods). Syllable clustering yielded 13 acoustic categories across recordings based on Deep Squeak tools. We compared the overall ΔF/F AUC between the left-Ctr and left-Wnt3 OE groups by pooling all syllables. No significant group difference was observed (**Figure S6C**, Wilcoxon rank-sum, $p > 0.05$). We then examined each of the 13 syllable categories individually, and none showed a significant difference between groups (**Figure S6D,** all $p > 0.05$). These results indicate that, under the present experimental conditions, Wnt3 overexpression in the left auditory cortex did not produce measurable changes in calcium activity amplitude both before and during vocalization.

To further elucidate the biological pathways through which Wnt3 overexpression in the left auditory cortex affects auditory feedback, we performed RNA sequencing on the left auditory cortex of Wnt3-OE and control mice. Differential expression analysis identified significant downregulation of genes associated with neuronal function in the left Wnt3-OE group (**Figure S7A**). Notably, these include Jun, a well-established negative regulator of myelination, and Opalin, an oligodendrocyte marker whose reduced expression may impair myelination. The downregulation of these genes was further validated by RT-qPCR, which confirmed significant lower expression levels of Jun and Opalin in the left Wnt3 OE group compared to controls (**Figure S7B**).

Pathway enrichment analysis suggest that Wnt3 overexpression may affect auditory feedback through mechanisms involving neuroinflammation, altered neuronal activity, or disrupted myelination (**Figure S7C**). In addition, gene set enrichment analysis (GSEA) revealed significant negative regulation of critical pathways,

including "Oxidative Phosphorylation" (adjusted P = 6.12e-6) and "MYC Targets V1" (adjusted P = 0.016), both essential for neuronal energy metabolism and synaptic plasticity (**Figure S7D-F**).

Together, Wnt3 overexpression in the left but not right auditory cortex altered USV spectral features and impaired myelination.

Related references

1. Dobin A, Davis CA, Schlesinger F, Drenkow J, Zaleski C, Jha S, et al. STAR: ultrafast universal RNA-seq aligner. Bioinformatics (Oxford, England). 2013;29(1):15-21. Epub 2012/10/30. doi: 10.1093/bioinformatics/bts635. PubMed PMID: 23104886; PubMed Central PMCID: PMC3530905.
2. Danecek P, Bonfield JK, Liddle J, Marshall J, Ohan V, Pollard MO, et al. Twelve years of SAMtools and BCFtools. GigaScience. 2021;10(2). Epub 2021/02/17. doi: 10.1093/gigascience/giab008. PubMed PMID: 33590861; PubMed Central PMCID: PMC7931819.
3. Pertea M, Pertea GM, Antonescu CM, Chang TC, Mendell JT, Salzberg SL. StringTie enables improved reconstruction of a transcriptome from RNA-seq reads. Nature biotechnology. 2015;33(3):290-5. Epub 2015/02/19. doi: 10.1038/nbt.3122. PubMed PMID: 25690850; PubMed Central PMCID: PMC4643835.
4. Love MI, Huber W, Anders S. Moderated estimation of fold change and dispersion for RNA-seq data with DESeq2. Genome biology. 2014;15(12):550. Epub 2014/12/18. doi: 10.1186/s13059-014-0550-8. PubMed PMID: 25516281; PubMed Central PMCID: PMC4302049.
5. Zhou Y, Zhou B, Pache L, Chang M, Khodabakhshi AH, Tanaseichuk O, et al. Metascape provides a biologist-oriented resource for the analysis of systems-level datasets. Nature communications. 2019;10(1):1523. Epub 2019/04/05. doi: 10.1038/s41467-019-09234-6. PubMed PMID: 30944313; PubMed Central PMCID: PMC6447622.
6. Wu T, Hu E, Xu S, Chen M, Guo P, Dai Z, et al. clusterProfiler 4.0: A universal enrichment tool for interpreting omics data. Innovation (Camb). 2021;2(3):100141. Epub 20210701. doi: 10.1016/j.xinn.2021.100141. PubMed PMID: 34557778; PubMed Central PMCID: PMC8454663.
7. Liberzon A, Birger C, Thorvaldsdóttir H, Ghandi M, Mesirov JP, Tamayo P. The Molecular Signatures Database (MSigDB) hallmark gene set collection. Cell Syst. 2015;1(6):417-25. doi: 10.1016/j.cels.2015.12.004. PubMed PMID: 26771021; PubMed Central PMCID: PMC4707969.
